# ERG is required for the differentiation of embryonic stem cells along the endothelial lineage

**DOI:** 10.1186/1471-213X-9-72

**Published:** 2009-12-23

**Authors:** Vesna Nikolova-Krstevski, Lei Yuan, Alexandra Le Bras, Preethi Vijayaraj, Maiko Kondo, Isabel Gebauer, Manoj Bhasin, Chris V Carman, Peter Oettgen

**Affiliations:** 1 Division of Cardiology, Beth Israel Deaconess Medical Center, Harvard Medical School, Boston MA 02215, USA; 2 Division of Molecular and Vascular Medicine, Beth Israel Deaconess Medical Center, Harvard Medical School, Boston MA 02215, USA; 3 Division of Interdisciplinary Medicine and Biotechnology, Beth Israel Deaconess Medical Center, Harvard Medical School, Boston MA 02215, USA; 4 Department of Medicine, and the Center for Vascular Biology Research, Beth Israel Deaconess Medical Center, Harvard Medical School, Boston MA 02215, USA

## Abstract

**Background:**

The molecular mechanisms that govern stem cell differentiation along the endothelial lineage remain largely unknown. Ets related gene (ERG) has recently been shown to participate in the transcriptional regulation of a number of endothelial specific genes including VE-cadherin (CD144), endoglin, and von Willebrand's Factor (vWF). The specific role of the ETS factor ERG during endothelial differentiation has not been evaluated.

**Results:**

ERG expression and function were evaluated during the differentiation of embryonic stem cells into embryoid bodies (EB). The results of our study demonstrate that ERG is first expressed in a subpopulation of vascular endothelial growth factor receptor 2 (VEGF-R2) expressing cells that also express VE-cadherin. During ES cell differentiation, ERG expression remains restricted to cells of the endothelial lineage that eventually coalesce into primitive vascular structures within embryoid bodies. ERG also exhibits an endothelial cell (EC)-restricted pattern during embryogenesis. To further define the role of ERG during ES cell differentiation, we used a knockdown strategy to inhibit ERG expression. Delivery of three independent shRNA led to 70-85% reductions in ERG expression during ES cell differentiation compared to no change with control shRNA. ERG knockdown was associated with a marked reduction in the number of ECs, the expression of EC-restricted genes, and the formation of vascular structures.

**Conclusion:**

The ETS factor ERG appears to be a critical regulator of EC differentiation.

## Background

Vasculogenesis, the development of the primary vasculature during embryogenesis, requires a highly orchestrated series of events that are spatially and temporally regulated [1]. Intra-embryonic vasculogenesis is preceded by extra-embryonic vascular development within the yolk sac [2]. Pioneering studies conducted in avian embryos and subsequently extended in amphibian and mammalian model systems in multiple different species have demonstrated the close association between the development of the hematopoietic and endothelial lineages [3].

The hemangioblast is a bipotent cell of mesodermal origin that can give rise to hematopoietic and ECs. The possible existence of a common precursor was originally suggested because of the close spatial association of hematopoietic cells and ECs in the blood islands associated with the developing embryos, and also because hematopoietic and ECs co-express a number of genes. One of the earliest markers expressed on cells of endothelial and hematopoietic origin is the VEGF receptor Flk-1 or VEGF-R2. Further support for the existence of the hemangioblast comes from the differentiation of embryonic stem cells along the endothelial and hematopoietic lineages [4,5]. When individual cells are allowed to differentiate further, they form adherent cells that express more endothelial specific markers whereas many of the non-adherent cells, presumed to be of hematopoietic origin, express such genes as β-hemoglobin [6]. Differentiation along the hematopoietic lineage is marked by the expression of cell surface markers including CD41 and CD45. A similar, time-dependent change in the expression of cell surface markers on cells of the hematopoietic and endothelial lineage occurs during the differentiation of embryonic stem cells into embryoid bodies.

Several receptor tyrosine kinases, including the VEGF-R2, VEGF-R1, Tie1, and Tie2 genes are known to be critical mediators of endothelial differentiation and vascular development. Targeted disruption of any of these genes leads to defects in vascular development and early embryonic lethality [7-9]. Comparison of the mouse and human DNA sequences within the regulatory regions of these genes has facilitated the identification of conserved binding sites for different classes of transcription factors. The Tie1 gene promoter contains conserved binding sites for ETS factors and AP2 [10]. Mutations in most of these conserved binding sites leads to marked reductions in the ability of the Tie1 promoter to direct LacZ gene expression in transgenic animals. A similar approach has been used to identify conserved binding sites for the ETS factors, SCL/tal-1, and GATA factors in the VEGF-R2 gene. Point mutations in some of these binding sites also leads to marked reductions in the vascular specific expression directed by the VEGF-R2 regulatory regions in transgenic studies [11]. Conserved ETS binding sites exist in the Tie2 and VEGF-R1 genes [12,13]. The results of these studies strongly support the hypothesis that members of certain transcription factor families, including the ETS and GATA transcription factor families are involved in regulating the different stages of vascular development and endothelial differentiation by regulating endothelial-specific gene expression.

The ETS genes are a family of at least thirty members that function as transcription factors. All ETS factors share a highly conserved 80-90 amino acid long DNA binding domain, the ETS domain [14]. Many macrophage, B cell and T cell specific genes are regulated by ETS factors. More recently several vascular-specific genes have been shown to be downstream targets of selected ETS factors. In addition to the Tie1, Tie2, VEGF-R1, and VEGF-R2 genes mentioned above, several other endothelial specific genes including von Willebrand Factor (vWF), PECAM-1, VE-cadherin, endothelial nitric oxide synthase genes have functionally important conserved binding sites for members of the ETS transcription factor family [15-18]. The ETS factor ERG has recently been shown to interact with a number of other transcription factors to regulate several endothelial-specific genes including angiopoietin-2, VE-cadherin, endoglin, and vWF [17-20]. The goal of this study was to determine the role of ERG as a transcriptional regulator of ES cell differentiation, and in particular, to determine whether it plays a role in regulating EC differentiation or hematopoiesis.

## Results

### ERG expression during endothelial differentiation

**Figure 1:**
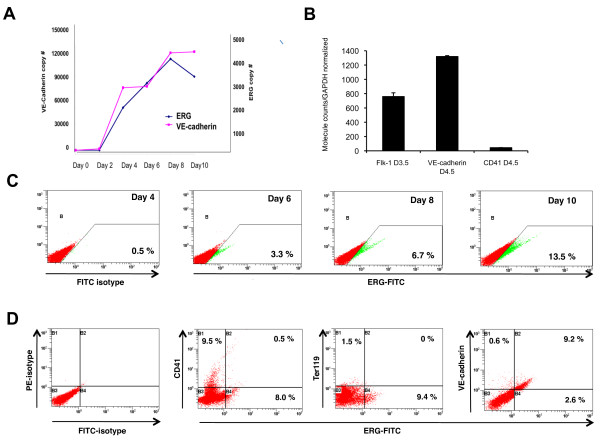
**ERG expression in differentiating EBs is endothelial-cell specific**. (**A**) Quantitative RT-PCR of RNA derived from EBs at different stages of differentiation (days 0-10), showing temporal correlation in the expression of ERG (blue) and endothelial-specific VE-cadherin (purple). Copy numbers (normalized per 10,000 copies GAPDH) are shown for VE-cadherin (left) and ERG (right) Y-axis. (n = 3) (**B**) ERG expression in sorted cell populations. Q-RT-PCR analysis of RNA isolated from EB cells sorted for VEGF-R2 at day 3.5, and VE-cadherin and CD41 at day 4.5 are shown. ERG is present in the mesodermal/hemangioblast cells expressing VEGF-R2 at day 3.5. ERG is highly enriched in the VE-cadherin expression population, but is almost absent in the CD41^+ ^cells at day 4.5. Error bars indicate means +/- S.D. (n = 3)(**C**) Flow cytometry analysis of ERG expression in EBs at different time points of ES cell differentiation. The increasing percentage of cells expressing ERG over time is shown by the shifting cell population (green) along the x-axis, compared to the ERG negative cells, shown in red. (**D**) Flow cytometry analysis of ERG expression in relation to other cell-lineage specific markers: hematopoietic (CD41 and Ter119), and endothelial (VE-cadherin) in day 8.5 EBs. Cell population expressing ERG was labeled with green fluorescent marker (FITC) and migrated along the x-axis.

We recently completed a gene profiling study comparing the expression of genes in VEGF-R2 expressing embryonic stem cells as they differentiate along the endothelial versus the hematopoietic lineages. Of particular interest to us was the ETS factor ERG which was restricted to VE-cadherin positive cells[21]. We analyzed ERG expression further during ES cells differentiation by quantitative (Q)-RT-PCR and observed a very similar temporal pattern for the expression of ERG and VE-cadherin (Figure 1A). Fluorescence activated cell sorting (FACS) was used to further define the specific populations of cells expressing ERG during ES cell differentiation. Cells expressing either VEGF-R2 (day 3.5), the endothelial-specific marker VE-cadherin (day 4.5), or the hematopoietic marker CD41 (day 4.5) were sorted and RNA was extracted from these cells. Q-RT-PCR demonstrated that ERG was expressed in VEGF-R2 positive cells at day 3.5 and then in VE-cadherin positive cells, but not CD41 expressing cells at day 4,5 (Figure 1B). Flow cytometry using an antibody directed against ERG demonstrated a time-dependent increase in ERG expression during differentiation with approximately 13.5% of cells expressing ERG by day 10 (Figure 1C). ERG segregation with specific cell populations (i.e. hematopoietic and/or endothelial) was evaluated at day 8.5 by flow cytometry using antibodies directed against ERG and one of the following: VE-cadherin, CD41 and the erythroid marker Ter119. ERG is predominantly expressed in VE-cadherin positive cells, and not CD41 or Ter119 positive cells (Figure 1D). Although only a small percentage of cells express Ter119 at day 8.5, ERG was not expressed in Ter119 expressing cells at later time points either (Additional file 1).

### Evaluation of temporal and spatial expression of ERG during ES cell differentiation

**Figure 2:**
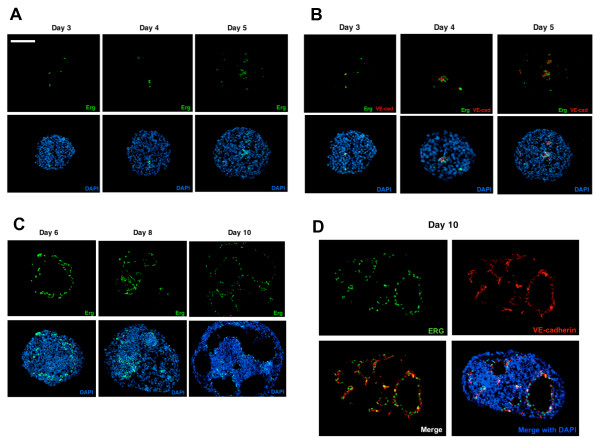
**Temporal and spatial ERG expression pattern in differentiating ES cells**. **(A) **Fluorescence immunostaining of frozen EB sections with rabbit anti-ERG and Alexa 498 anti-rabbit IgG (green) at the early stages of EB differentiation. ERG is first detected in the developing EBs at day 3. **(B) **Double immunostaining of frozen EB sections with ERG Ab (green) and VE-cadherin Ab (red) shows consistent co-localization of the two markers during the time-course of EB differentiation. DAPI nuclear staining (shown in blue) provides an outline of the general EB morphology. **(C) **Fluorescence immunostaining of frozen EB sections with anti-ERG Ab (green) at later stages of EB differentiation (days 6, 8, and 10) demonstrates ERG expression and organization in more complex vascular structures lining the walls of the vascular channels. **(D) **Co-expression of ERG (green) and VE-cadherin (red) in the vascular channels of day 10 EB. DAPI nuclear staining (blue) provides an outline of the general EB morphology. The images were obtained with Leica fluorescence microscope at 40× magnification. Scale bar represents 200 μm.

ERG and VE-cadherin expression were evaluated in frozen EB sections at day 3-10 by immunofluorescence to define the temporal and spatial pattern of ERG expression in developing EBs. ERG and VE-cadherin are first expressed at day 3 to 4, and there is a very close association between cells expressing ERG (green) and those expressing VE-cadherin (red) throughout EB differentiation (Figure 2A, 2B). Whereas ERG appears to be predominantly restricted to the nucleus of cells, VE-cadherin is observed on the cell surface. Furthermore, VE-cadherin expressing cells coalesce into structures resembling primitive blood vessels and line the inner surface of the cystic structures (Figure 2C, 2D). Further demonstration of the close association between the VE-cadherin expressing cells and ERG was observed by confocal microscopy also demonstrating the nuclear localization of ERG (Additional file 2). A similar close association between ERG and another EC-restricted protein endoglin (CD105) was also observed during ES cell differentiation (Additional file 3), but that was distinct from the hematopoietic marker CD45.

### ERG expression during vascular development

**Figure 3:**
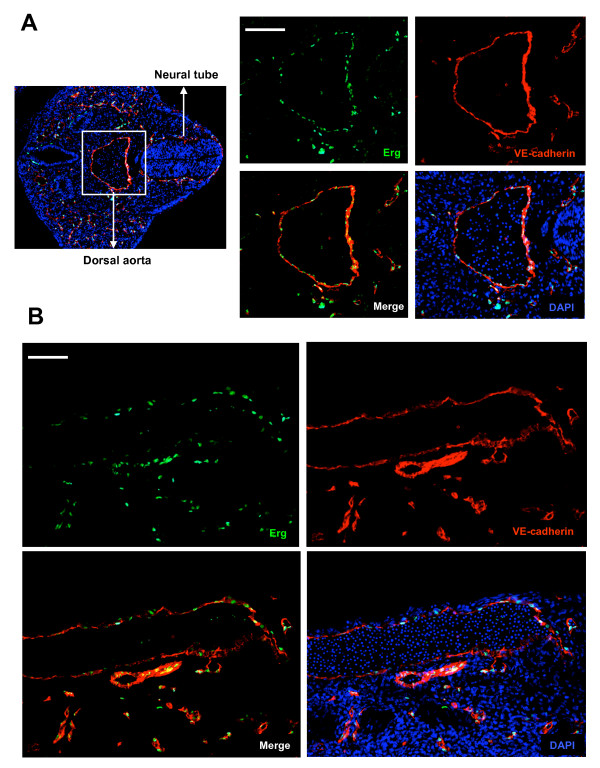
**ERG and VE-cadherin expression in E9.5 and E12.5 embryos**. **(A)**Transverse section of an E9.5 embryo (left). Right hand 4-part panel demonstrates ERG expression (green), VE-cadherin (red); overlap of ERG and VE-cadherin (lower left) and with DAPI staining (lower right). **(B) **Evaluation of ERG (green) and VE-cadherin (red) expression in sagittal sections of the dorsal aorta (E12.5) of the mouse embryo. Co-localization of ERG and VE-cadherin are shown in the lower left hand panel, and with DAPI nuclear staining (bottom right panel). The images were obtained with Leica fluorescence microscope at 10× and 40× magnification. Scale bars: 70 μm (A) and 40 μm (B).

**Figure 4:**
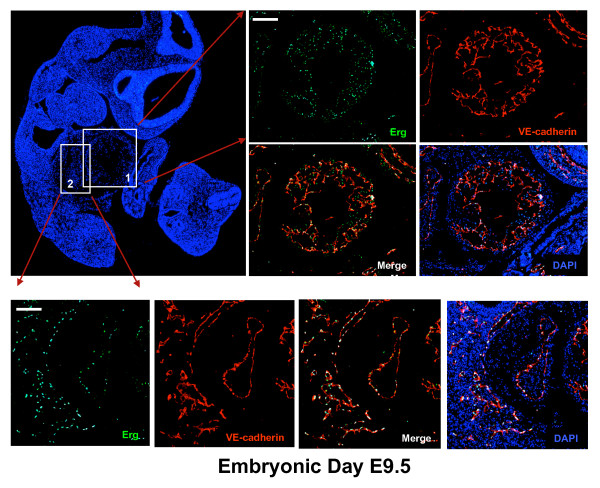
**Evaluation of ERG expression in the developing heart**. Sagittal sections of E9.5 mouse embryos with DAPI staining (left upper panel). High power magnification of the developing cardiac ventricular chamber (upper right) and common atrial chamber. The ventricular chamber is highly trabeculated and expression of the endocardial surface is shown by VE-cadherin staining (red). ERG (green) co-localizes with VE-cadherin expressing cells in both the atrium and ventricular chambers. The images were obtained with Leica fluorescence microscope at 10× and 40× magnification. Scale bars represent 80 μm and 70 μm (top and bottom panel, respectively).

To begin to define ERG expression during vascular development, mouse embryos at different stages of development were isolated. ERG expression was evaluated by immuno-fluorescence. As early as E7.5 ERG and VEGF-R2 appear to be expressed in the blood islands enriched in hemangioblasts that are the common early precursors for the endothelial and hematopoietic lineages. Serial sections of yolk sac demonstrate expression of ERG and VEGF-R2 in the same region (Additional file 4). Later during development, we observed a close correlation between ERG and VE-cadherin expression in developing vascular structures and blood vessels in a number of tissues. For example, ERG expression is observed in ECs of the dorsal aorta at embryonic day 9.5 (Figure 3A), and at day 12.5 (Figure 3B). Although there is faint ERG staining in a few cells within the lumen of the aorta, the vast majority of ERG staining is located within the vessel wall in close approximation with VE-cadherin. Just as in the EBs, ERG expression is localized within the nucleus of VE-cadherin expressing cells. ERG expression is also observed in the trabeculated endocardial surface of the common atrium and ventricle of the heart at embryonic stage E9.5 (Figure 4). EC-specific expression was also observed in several other tissues including the intersomitic, and mesonephric blood vessels of the aorta-gonado-mesonephros (AGM) region, and blood vessels of the umbilical cord (Additional files 5, 6, and 7).

### Lentiviral knockdown of ERG in ES cells

**Figure 5:**
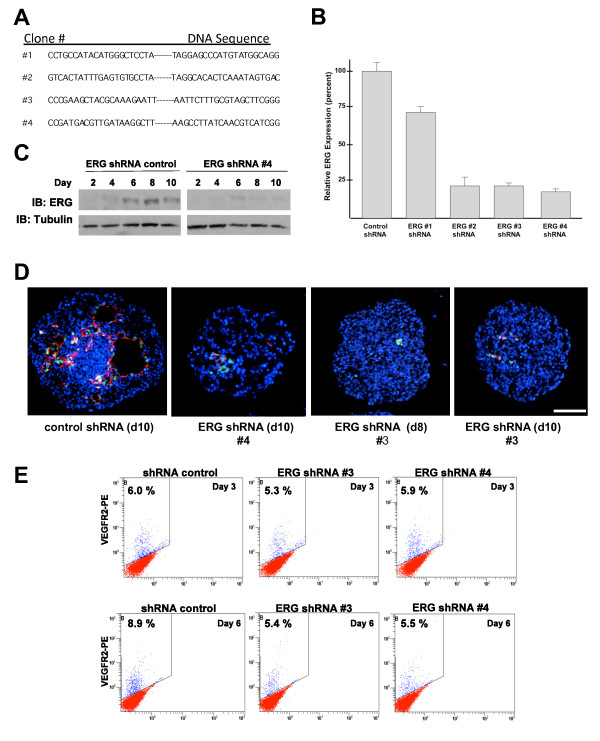
**ERG knockdown in ERG shRNA treated EB**. (**A**) Four different ERG shRNA sequences were used for transduction and expression in ES cells prior to EB differentiation. A scrambled nucleotide sequence was used as a control. (**B**) Evaluation of ERG expression by Q-RT-PCR in EBs at day 8 using lentivirally delivered control, and ERG shRNAs (lentivirally delivered sequences #1-4 were used to generate ERGshRNA ES cell clones #1-4 respectively). Expression levels are shown as a percentage of control shRNA treated cells Error bars indicate means +/- S.D. (n = 4). (**C**) Evaluation of ERG expression in differentiating control and ERG shRNA treated ES cells by Western blot analysis. (**D**) Immunohistochemical analysis of ERG (green), VE-cadherin (red), and nuclear staining DAPI (blue) in EBs from control and ERG shRNA clones # 3 and 4 at day 10 and day 8 (# 3) The images were obtained using Leica fluorescence microscope at 40× magnification. Scale bar represents 100 μm. (**E**). Evaluation of VEGF-R2 expression in ERG shRNA (#3 and #4), or control shRNA treated ES cells by flow cytometry at 3 and 6 days after differentiation.

To further define the role of ERG as a transcriptional regulator of endothelial-specific gene expression and endothelial differentiation, we tested the ability of four different lentiviral vectors encoding shRNA directed against mouse ERG to suppress ERG expression during ES cell differentiation. Sequence analysis of the shRNA sequences confirmed the specificity for ERG (with no significant sequence homology to other ETS family members). Undifferentiated murine CCE ES cells were infected with control (scrambled shRNA) and the four lentiviral vectors directed against ERG (Figure 5A). Puromycin was used to stably select individual clones. ERG expression was measured in the clones 8 days after their differentiation into EBs. A 75-80% reduction in ERG expression was observed in three out of the four ERG shRNA (clones 2, 3, and 4), as measured by Q-RT-PCR and Western blot analysis when compared to control shRNA (Figure 5B and 5C). To evaluate whether changes in ERG expression are associated with alterations in the number of ECs and/or vascular structure formation, we examined the expression of ERG and VE-cadherin in the ERG shRNA clones. Whereas the control shRNA clones exhibited a similar pattern of vascular structure formation to untransfected ES cells during EB formation, there was a marked reduction in the number of VE-cadherin expressing cells and the formation of vascular structures in the ERG shRNA clones (Figure 5D). Because we first observed ERG expression in a subset of VEGF-R2 expressing cells, we also wanted to determine whether ERG affects the number of VEGF-R2 expressing cells or hemangioblast development. VEGF-R2 expression was evaluated in two of the ERG knockdown clones compared to the control clones. There was no change in the number of VEGF-R2 expressing cells at day 3 (Figure 5E), suggesting that ERG does not regulate hemangioblast development, but rather the very early stages of EC differentiation. At later time points (day 6), the number of VEGF-R2 positive cells decreases in ERG shRNA EBs as compared to controls which is most likely due to the decreased number of EC progenitors or ECs expressing VEGF-R2 (Figure 5E).

### Flow cytometric evaluation of ERG and control shRNA treated ES cells

To evaluate the effect of ERG suppression on endothelial and hematopoietic lineages, flow cytometric analysis using markers specific for both lineages was performed on day 6, 8 and 10 of differentiation. A reduction in several genes expressed on endothelial cells was observed in the ERGshRNA clones compared to the controls, with the most striking reductions in the direct targets of ERG VE-Cadherin and endoglin, particularly at day 10 (Table 1 and Additional file 8) [19,22]. It is known that some of these genes, such as endoglin are expressed on the hemangioblast cells as well as endothelial cells [23]. In a separate experiment using shRNA knockdown of ERG in HUVECS cells, we identified that Tie2 and VEGF-R1 (Flt-1) are not direct targets of ERG (data not shown). However, a significant reduction of Tie2 and VEGF-R1 was seen in our ERGshRNA clones by FACS analysis, indicating that suppression of ERG affects not only the expression of ERG target genes but also the number of ECs. In contrast, no change in the expression of CD41 and CD45 occurred suggesting that there was no significant effect on hematopoietic lineage differentiation (Table 1). Furthermore, we observed a decrease of about 30% in the potential of the ERGshRNA clones to form vascular sprouts when compared to the controls (Additional file 9). This may be related to a decrease in the number of ECs and/or impaired function of the ECs due the reduction in the expression of several endothelial-restricted genes. We also evaluated the ability of control and ERGshRNA ES cells to form hematopoietic cells using a blast colony-forming cell assay. No difference was observed in the hematopoietic potential of control versus ERGshRNA ES cells (Additional file 10).

**Table 1:** FACS analysis of control and ERG shRNA treated embryoid bodies.

	Day 6		Day 8		Day 10	
			
	Control	ERG ShRNA	Control	ERG ShRNA	Control	ERG ShRNA
**CD 41**	1.4%	1.9%	2.5%	2.1%	2.3%	5.5%
**CD 45**	0.8%	1.3%	0.7%	0.5%	2.4%	3.1%
**VE-Cadherin**	5.8%	1.5%	19.3%	10.4%	26.8%	5.7%
**Endoglin**	23.6%	1.8%	26.0%	19.3%	25.7%	10.2%
**Tie2**	10.5%	9.3%	19.6%	17.4%	20.6%	13.2%
**VEGF-R1**	26.6%	25.9%	34.9%	25.4%	21.8%	16.4%

Flow cytometric analysis of control and ERG shRNA treated EBs at different time points (day 6, 8, 10) using antibodies directed against endothelial (VE-cadherin, endoglin, Tie2, and VEGF-R1) and hematopoietic (CD41, CD45) cell surface markers.

Identification of ERG downstream targets during ES cell differentiation

**Figure 6:**
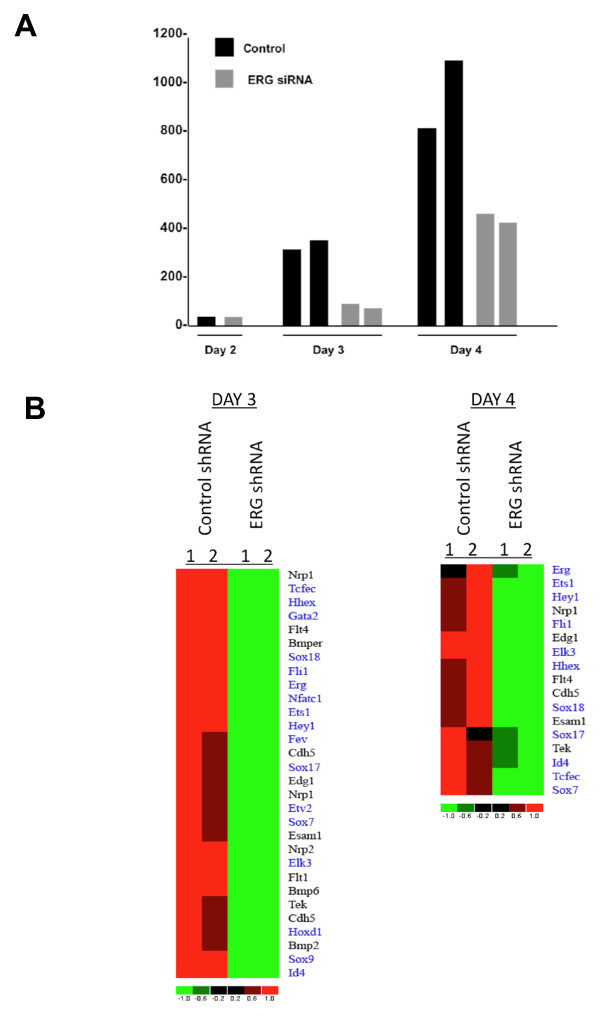
**ERG target genes in VEGF-R2^+ ^ES cells**. (**A**) Normalized microarray expression values for ERG in VEGF-R2^+ ^cells sorted from ERG shRNA or control cells 2, 3, and 4 days after differentiation. (**B**) Heat map of a subset of genes that are significantly differentially downregulated after 3 days and 4 days in ERG shRNA versus control ES cells. In the heatmaps, rows represent genes and columns represent samples, ordered as control and shRNA treated. Genes are clustered using row-normalized signal values which are mapped to the [-1,1] interval. Transcription factors are shown in blue. All other genes are shown in black. Signal values are color-coded so that red and green represent high and low expression values, respectively.

Previous studies have shown that targeted disruption or knockdown of VE-cadherin alone is not sufficient to inhibit EC differentiation [24]. To further define the molecular mechanisms by which ERG regulates EC differentiation, we sorted VEGF-R2 expressing cells after 2, 3, and 4 days of differentiation using mouse ES cells with stable suppression of ERG and from control cells. ERG expression was nearly undetectable at day 2 (Figure 6A). Early expression was detected at day 3 and significant reductions of ERG expression were observed in the ERG shRNA cells at both day 3 and day 4. Comparison of ERG shRNA and control shRNA cells at day 3 and day 4 revealed several genes that were differentially expressed (LCB >1.5). Most of the genes were repressed upon ERG suppression. The heat map of a selected number of differentially downregulated genes at day 3 and 4 are shown (Figure 6B and Additional file 11). We observed reductions in the expression of a number of transcription factors as well as EC-restricted genes. Quantitative RT-PCR was used to evaluate changes in the expression of selected ETS transcription factors in differentiated ES cells derived from control and ERG shRNA treated clones. Whereas a significant reduction in the expression of ERG was observed, no change in the expression of other Ets factors was observed (Additional file 12A). We similarly examined the expression of a number of mesodermal and hematopoietic markers and did not observe significant changes in the expression of these markers, with the exception of the Scl gene where we observed a slight reduction (Additional file 12B). In contrast, reductions in several EC-restricted proteins and transcription factors known to regulate EC-restricted gene expression were observed (Additional file 12C). Further validation of some of the putative ERG targets was evaluated by chromatin immunoprecipitation (Additional file 13). In addition to VE-cadherin, we confirmed binding of ERG to putative ERG binding sites within the proximal promoters of the Hey2 and endoglin genes.

## Discussion

The results of several recent studies point towards an emerging role for ERG as a transcriptional regulator of EC-restricted genes [17-20]. The findings of our study support a unique role for the ETS factor ERG as a transcriptional regulator of endothelial differentiation. ERG expression was first detected in a sub-population of VEGF-R2+ expressing cells that co-express VE-cadherin, and closely correlates with the expression of VE-cadherin during ES cell differentiation. Inhibition of ERG expression, however, did not alter the number of VEGF-R2 expressing cells just prior to their differentiation along the endothelial and hematopoietic lineages suggesting that ERG is not required for the formation of the hemangioblast or hematopoiesis. A recent genome-wide analysis of ETS factors in zebrafish identified three members of the ETS transcription factor family, including ERG, Fli-1, and ETSRP, as playing a role in hemangioblast differentiation and angiogenesis [25]. Of these ETS family members, ETSRP was expressed earliest and required for hemangioblast formation. Similarly, mice that have a mutation in the ER71, the homologue of ETSRP exhibit defects in hematopoiesis and endothelial differentiation [26]. In contrast, neither knockdown of ERG or Fli-1 in zebrafish led to abnormalities in hemangioblast formation, which is consistent with our findings in murine ES cells.

We observed a very close correlation of ERG and VE-cadherin expression in the EB throughout ES cell differentiation. ERG expression also closely correlates with VE-cadherin expression during embryonic vascular development. ERG has previously been shown to regulate the VE-cadherin gene in ECs [22]. Targeted disruption of VE-cadherin prevented the formation of vessel-like structures in embryoid bodies [27]. During mouse development, deficiency of VE-cadherin did not affect the initial assembly of a vascular plexus but was associated with impaired vascular remodeling and maturation [24]. Knockdown of ERG expression not only affected vascular structure formation in embryoid body, but was also associated with a marked reduction in the number of EC. This suggests that ERG must regulate additional genes involved in the formation of EC. Similar knockdown studies were conducted in zebrafish using morpholino oligonucleotides (MO). MOs directed against ERG resulted in hemorrhagic defects in the brain between days 2.5 and 3.0 without any obvious morphological defects [25]. Similar defects were observed with MOs directed against Fli-1. Double knockdown of ERG and Fli-1 led to more severe developmental defects with disorganization of the intersomatic vessels. In contrast to our study, knockdown of ERG in zebrafish was not associated with a significant reduction in the number of ECs that would be expected to be associated with more pronounced defects in vascular development. One possible explanation for the differences observed is that the MOs may have reduced, but not abolished the levels of ERG to a point where sufficient levels of the ERG protein were present to promote EC differentiation but not later stages of vascular development. Alternatively, changes in the function of selected ETS family members may have occurred over the course of evolution, with ERG playing a more dominant role in murine vascular development than in zebrafish.

One of the earliest known markers of hematopoiesis is CD41 (glycoprotein IIb) [28]. While CD41 expressing ES cells are capable of contributing to multiple hematopoietic lineages, they cannot differentiate into ECs. We did not observe significant expression of ERG in VEGF-R2+ cells expressing CD41. Furthermore, ERG knockdown was not associated with reductions in hematopoietic progenitor cells. This is in contrast to recent reports supporting a role for ERG in hematopoiesis [29]. A missense mutation associated with the alteration of one amino acid in the ERG DNA binding domain *(Mld2) *was associated with marked defects in hematopoiesis due to reductions in transactivation but no change in DNA binding affinity. The results of this study contrast with our knockdown studies, and might be explained by the fact that a mutation in ERG could result in the formation of a dominant-negative protein with altered affinity for different target genes or changes in the affinity of ERG for different interacting proteins.

One of the major downstream targets of ERG is VE-cadherin. Within the developing embryo there are also temporal changes in the expression of VE-cadherin in hematopoietic stem cells (HSCs) [30]. Whereas VE-cadherin is expressed in HSCs within the AGM of embryos at day 11.5, and in the yolk sac at day 12.5, there is marked down regulation of VE-cadherin expression during fetal liver colonization and complete loss in HSCs with the adult bone marrow formation, suggesting that although a subset of HSCs may be derived from a common bipotent precursor population, expression of endothelial markers is not required for maintenance of long-term HSC progenitor cells at later stages of embryonic development or in the adult. Previous studies have demonstrated that VEGF-R2+/CD144+ isolated from mouse embryos 7.5 to 9.5 days post coitum (dpc) or from human ES cells that are CD45 negative but are capable of differentiating along the endothelial and hematopoietic lineages [31,32]. Although VE-cadherin expressing cells are capable of differentiating into hematopoietic cells, VE-cadherin negative cells exhibited a 15-20 fold greater potential of promoting definitive hematopoiesis than did those expressing VE-cadherin [33]. Targeted disruption of the VE-cadherin gene did not significantly impair hematopoiesis, with similar numbers of erythroid, myeloid, and mixed hematopoietic progenitor cells in E8.5 embryos compared to wild type controls [24]. This would support the overall concept of a "hemogenic" endothelium, but with a predominant mechanism of hematopoietic differentiation being independent of EC differentiation.

ERG is among a very few number of transcription factors that exhibit an EC-restricted expression pattern. For example, Vezf1 is a zinc finger transcription factor that contributes to vascular remodeling and the development of both the vascular and lymphatic endothelium [34]. The homeobox transcription factor *Hex *is expressed at the hemangioblast stage and contributes to the hematopoietic and endothelial lineages [34]. Another family of transcription factors belonging to the FoxO subset of forkhead transcription factors are expressed in ECs and function as transcriptional regulators of angiogenic growth factors and vascular stabilization [35]. Selected transcription factors have also been identified that regulate later stages of endothelial differentiation, into arterial (Hey1 and Hey2), venous (COUP-TFII), and lymphatic endothelium (Prox-1) [35]. ERG shRNA was associated with significant decreases in the expression of Vezf1, Hey1, and Hey2, with confirmation of at least one of these transcription factors, Hey2, as being a direct target of ERG as demonstrated by ChIP. In contrast, transcription factors known to be critical regulators of hematopoiesis, including Runx1 and HoxB4, were not significantly altered by knockdown of ERG. In summary, the results of our study provide strong evidence for a critical role for ERG as a transcriptional regulator of early EC differentiation that does not affect hematopoiesis or hemangioblast development.

## Conclusion

The results of our study demonstrate that ERG regulates the expression of a number of EC-restricted genes during ES cell differentiation. Knockdown of ERG is associated with a significant reduction in the formation of vascular structures in developing embryoid bodies and the number of endothelial cells, that may have important implications for the role of ERG in vascular development during mouse embryogenesis. ERG does not appear to be required for the development of hematopoietic precursor cells during ES cell differentiation. ERG may therefore play a unique role as a transcriptional regulator of EC differentiation that is distinct from hematopoiesis.

### Methods (abbreviated; see Supplemental methods, additional file 14)

#### ES culture

CCE mouse embryonic stem cells (ATCC) were maintained on irradiated primary mouse embryonic fibroblasts (MEFs) (Chemicon) in knockout KO-DMEM (Invitrogen/Gibco-BRL) supplemented with ES-cell grade 15% fetal bovine serum (Hyclone, Logan, UT), penicillin/streptomycin 1% (Invitrogen/Gibco-BRL), L-glutamine 2 mM (Invitrogen/Gibco-BRL), non-essential amino acids 0.1 mM, nucleosides 0.1 mM, 2-mercaptoethanol 0.1 mM (Sigma, St. Louis, MO), and ESGRO leukemia inhibitory factor (LIF) 1000 units/ml (Sigma). ES cells were grown on MEFs in the presence of LIF to maintain their undifferentiated state for 48 hours.

#### Embryoid Body Model

The embryoid body is a widely accepted model of differentiation that recapitulates many of the early events of embryogenesis including hematopoiesis and endothelial differentiation. Prior to differentiation, the ES cells are removed from the feeder cells by trypsinization, mixed to a single cell suspension and plated at density of 2 × 10^6 ^cells/10 cm dish (Fisher Scientific) containing KO-DMEM growth media without LIF to promote ES differentiation.

#### Real Time PCR

SYBR Green I-based real-time PCR was carried out on an Opticon Monitor (MJ Research, Inc., Waltham, MA). All PCR mixtures contained PCR buffer with final concentration: 10 mM Tris-HCl (pH 9.0), 50 mM KCl, 2 mM MgCl2, and 0.1% TritonX-100), 250 μM deoxy-NTP (Roche), 0.5 μM of each PCR primer, 0.5× SYBR Green I, 5% DMSO, and 1 U Taq DNA polymerase with 2 μl cDNA in a 25 μl final volume of reaction mix. For each run, serial dilutions of human GAPDH plasmids were used as standards for quantitative measurement of the amount of amplified cDNA and GAPDH primers were used to measure the amount of GAPDH cDNA. For normalization of each sample, copy number was determined/10,000 copies of GAPDH in the sample.

#### Lentiviral shRNA for knockdown studies in ES cells

Lentivirus encoding the shRNA directed against particular gene targets of interest (Sigma) and polybrene (8 μg/ml; Sigma, Cat.# H9268) was added to the ES cells and incubated for 4 hours, after which 1 ml of ES media was added to the infected cells. Stable clones were puromycin selected and expanded.

#### Flow cytometry and Fluorescence-activated cell sorting (FACS)

EBs were washed twice in PBS (without Ca^2+ ^and Mg^2 ^and broken down into a single cell suspension. Flow cytometry runs and FACS sorting were performed on the FX5000 Flow Cytometer/FACS sorter at the Beth Israel Medical Center Flow Cytometry Facility, using the CXP analysis software.

#### Microarray analysis

For transcriptional profiling, the mouse genome 430 2.0 Affymetrix GeneChip, containing more than 45,000 transcripts were used. RNAs for the microarray experiments were obtained in duplicates from two separately conducted experiments using the murine embryonic stem cells.

## Authors' contributions

VN contributed to the writing and the majority of the ES cell experiments. LY contributed to the studies involving lentiviral knockdown of the ES cells, and ChIP studies. ALB contributed to the ES cell culture and flow cytometry studies. PV contributed to the EC sprouting assays. MK contributed to the quantitative RT-PCR experiments. IG contributed to the ES cell culture experiments. MB contributed to the bioinformatic experiments. CC contributed by making the confocal images. PO contributed to the overall design of the experiments and writing of the manuscript. All authors have read and approved the final manuscript.

## Supplementary Material

Additional file 1 **Analysis of ERG and Ter119 expression by flow cytometry during ES cell differentiation**. Expression of ERG (FITC labeled) and Ter119 (PE-labeled) was analyzed using the flow-cytometry technique in day 8.5, 10.5 and 12.5 EBs. Flow-cytometry diagrams show expression of the two markers on clearly two distinct cell populations (Ter199 population migrating along the y-axis, while the ERG population shifts along the x-axis). Click here for file

Additional file 2 **Evaluation of ERG expression by confocal microscopy**. EB sections were stained for ERG (green), VE-cadherin (red), and nuclei (blue) and visualized by laser-scanning confocal microscopy. A-C. ERG **(A)**, VE-cadherin **(B) **and merge of all three stains is depicted in **(C)**. **(D) **Depicts a subregion as indicated in panel C. **(E-G) **These panels depict a subregion of panel D as indicated, and show ERG **(E) **and nuclei **(G) **alone or merged **(F)**. Note that ERG staining is largely coincidental with nuclei. Scale bar = 100 μm in panel A; = 20 μm in panel D, and; = 5 μm in panel G. Click here for file

Additional file 3 **Fluorescence microscopy evaluation of ERG expression with endoglin and CD45 in day 10 EBs**. Panel **(A) **and **(B) **show double fluorescence immunostaining of ERG (green) with the endothelial-specific endoglin (red) and hematopoietic cell marker CD45 (red), respectively. Panel (A) demonstrates ERG co-localization with endoglin in the endothelial cells that line the vascular channel walls of the cystic EBs, while in panel (B), CD45 expression is seen in hematopoietic progenitor cells that bud off from the channel's wall and migrate towards the center of the vascular channel lumen. Scale bar = 100 μm. Click here for file

Additional file 4 **ERG and VEGF-R2 expression in E7.5 embryo**. (**A-B**)Serial sections of mouse E7.5 embryo showing expression of ERG and VEGF-R2 in a similar subset of cells in the blood islands, respectively. Arrowheads indicate nonspecific staining of the antibodies. (C, D) Higher magnification of (A, B). Arrows point to the cells of the blood islands that express ERG within the nucleus (C) and VEGF-R2 on the surface (D) al, allantois; am, amnion; bi, blood island; m, maternal decidua. Scale bars: 50 μm Click here for file

Additional file 5 **ERG expression in intersomatic blood vessels in the developing embryo at E12.5**. Sagittal sections of frozen mouse embryo at E12.5 (far left image) fluorescence stained with DAPI nuclear stain (blue). The inset box points to the developing somite region in the mouse embryo, which is 20× magnified in the middle column (a, c and e) and 40× magnified in the far right column (b, d and f). Both columns demonstrate ERG co-localization (a, b, green) with the endothelial-specific VE-cadherin (c, d, red) in the developing intersomatic blood vessels. Image (e) and (f) are overlays with DAPI (blue) which outlines the general tissue morphology. Images were obtained with Leica fluorescent microscope. Scale bars: 80 μm (a, c and e) and 30 μm (b, d and f). Click here for file

Additional file 6 **ERG expression in the aorta-gonado-mesonephros (AGM) region of the developing mouse embryo at E12.5**. **(A) **Sagittal section of a frozen mouse embryo at E12.5, fluorescence stained with DAPI nuclear stain (blue). The inset boxes 1 and 2 outline the AGM and umbilical cord remnant, respectively, in the E12.5 mouse embryo. Panel **(B) **represents magnification of inset 1 and shows ERG co-expression (a, green) with the endothelial-specific VE-cadherin (b, red) in the developing vasculature of the embryonic AGM region. (c) is a merged image of (a) and (b), while (d) is an overlay with DAPI (blue). The thin arrows in (c) point to the mesonephric blood vessels of the AGM region, while the thick arrow in (d) points to the dorsal (posterior) root ganglion of AGM. Images in panel (A) and (B) were taken with Leica fluorescent microscope at 10× and 20× magnification, respectively. Scale bar = 30 μm. Click here for file

Additional file 7 **ERG expression in the umbilical cord of day E12.5 embryo**. **(A) **Magnification of inset box 2 from Additional file 6 showing the DAPI stained remnants of the placenta and umbilical cord vein, respectively. ERG (green, a and e) also co-localizes with VE-cadherin (red, b and f) in the umbilical cord arteries (panel **B**) and umbilical cord vein (panel **C**). Scale bar represents 90 μm. Click here for file

Additional file 8 **Effect of ERG knockdown on its direct targets by flow cytometry analysis at different time points**. Flow cytometry analysis of the ERG target genes VE-cadherin and endoglin during EB differentiation in ERG shRNA control and shRNA #4 cells. Each flow cytometry diagram is labeled with PE-conjugated antigen-specific Ab that migrates along the y-axis. Click here for file

Additional file 9 **The effect of ERG knockdown on in-vitro vascular sprout formation on differentiating EB's**. **(A)**. Comparison of mean total length of vascular sprouts in control and ERG ShRNA knockdown EB's. Error bars indicate means +/- S.D. (n = 2, with 50-100 EBs per experiment) (**B**). Representative examples of EBs stained with CD31. Scale bar, 100 μm. Click here for file

Additional file 10 **Hematopoietic colony forming assay in control and ERG shRNA treated ES cells**. (**A**) Hematopoietic colonies including primitive erythroid colony Ery-P (a and b), blast colony forming unit containing definitive erythroid progenitors BFU-E (c and d), and colony forming units with granulocytes and macrophages CFU-GM (e and f) were formed by both, control shRNA ES cells (a, c, and e) and ERG shRNA treated ES cells (b, d, and f), when grown on methylcellulose base and in presence of hematopoietic growth factors. Images were taken on Nikon light microscope at 10× and 20× magnification. (**B**) Hematopoietic potential of control and ERG shRNA treated ES cells. The number of individual colonies (Ery-P, BFU-E, and CFU-GM) is expressed as percentage of the total number of colonies for each phenotype (control vs. ERG shRNA ES cells). (**C**) Values used in the table represent average number of counted colonies from three individual plates (n = 3). Error bars indicate means +/- S.D. Click here for file

Additional file 11 **List of differentially expressed genes in ERG shRNA versus control ES cells**. Selected list of genes that are significantly changed after 3 days or 4 of ES cell differentiation in the control versus ERG shRNA treated cells. The Fold change (FC) and Lower Bound of Fold change (LCB) of significantly changing genes is shown in BOLD. Click here for file

Additional file 12 **Real-time RT-PCR analysis of control and ERG shRNA treated EBs at day 4.5**. (**A**) Expression of ETS family of transcription factors in control shRNA and ERG shRNA treated EBs at day 4.5. (**B**) QRT-PCR evaluation of expression of mesodermal and hematopoietic markers in control and ERG shRNA treated EBs at day 4.5 of differentiation. (**C**) Analysis of expression of different endothelial-cell specific markers in control and ERG shRNA treated EBs at day 4.5. In all three experiments differential expression of cell-lineage specific markers in control EBs was compared with EBs treated with two different ERG shRNAs: sequence #3 and #4. For each molecular marker tested n = 3 and the error bars indicate means +/- S.D. Click here for file

Additional file 13 **Downstream targets of ERG**. (**A, B, C**) Top: Schematic diagram of ERG binding sites in VE-cadherin, Hey-2, or Endoglin promoter, respectively. The 1.5 kb upstream promoter region of each gene was analyzed in search for the potential ERG binding sites indicated with red boxes. The bidirectional arrows marked the target regions for ChIP assays (ChIP1 and/or ChIP2). (A, B, C) Bottom: ChIP assay using HUVEC. An ERG polyclonal antibody was used for precipitation. PCR analysis of the input, in the absence of ERG antibody (CTR), and in the presence of ERG antibody (ERG) after immunoprecipitation (IP) using primers corresponding to indicated ERG putative binding sites (ChIP1 and/or ChIP2) of the each gene promoter. Molecular weight markers are shown on the left. Click here for file

Additional file 14 **Supplemental Methods**. Supplemental methods not described in the main document. Click here for file
