# Supplementary figures and images for: ERG is required for the differentiation of embryonic stem cells along the endothelial lineage
Source: BMC Dev Biol. 2009 Dec 23;9:72. doi: 10.1186/1471-213X-9-72 (PMC2803788; doi:10.1186/1471-213X-9-72)

**D 8.5**

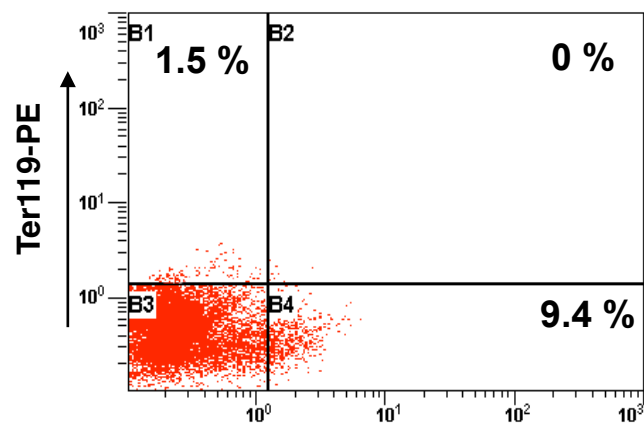

**D 10.5**

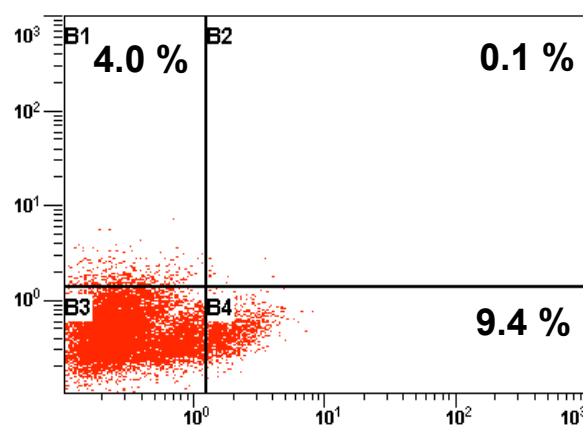

**D 12.5**

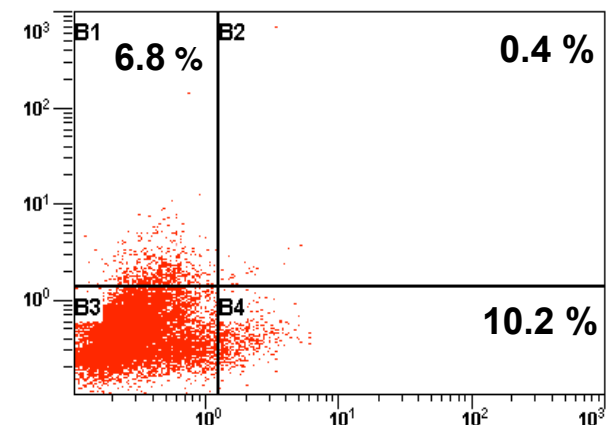

**ERG-FITC**

Supplement: Additional file 1 — Analysis of ERG and Ter119 expression by flow cytometry during ES cell differentiation. Expression of ERG (FITC labeled) and Ter119 (PE-labeled) was analyzed using the flow-cytometry technique in day 8.5, 10.5 and 12.5 EBs. Flow-cytometry diagrams show expression of the two markers on clearly two distinct cell populations (Ter199 population migrating along the y-axis, while the ERG population shifts along the x-axis). [file 1471-213X-9-72-S1.PDF]

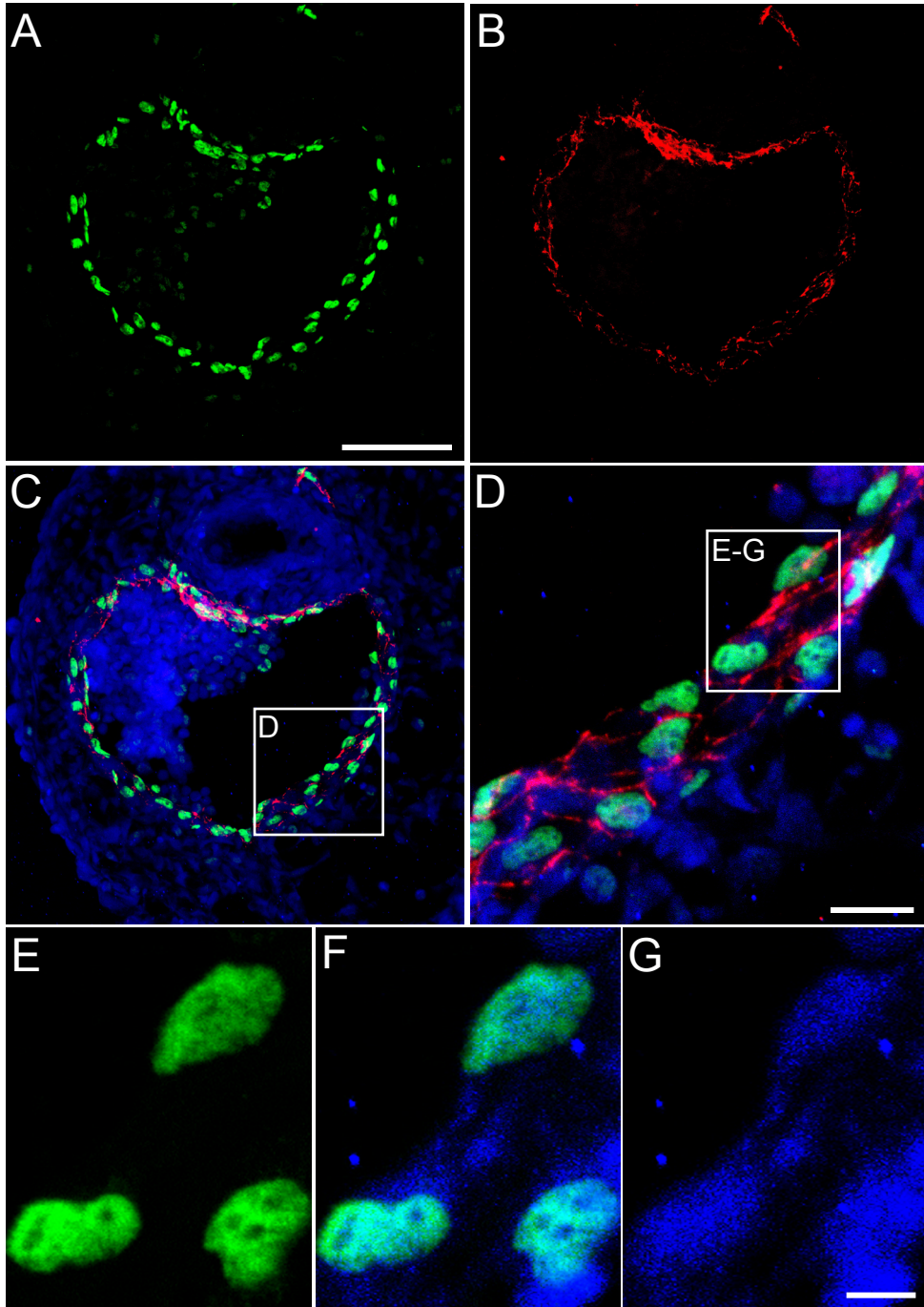

Supplement: Additional file 2 — Evaluation of ERG expression by confocal microscopy. EB sections were stained for ERG (green), VE-cadherin (red), and nuclei (blue) and visualized by laser-scanning confocal microscopy. A-C. ERG (A), VE-cadherin (B) and merge of all three stains is depicted in (C). (D) Depicts a subregion as indicated in panel C. (E-G) These panels depict a subregion of panel D as indicated, and show ERG (E) and nuclei (G) alone or merged (F). Note that ERG staining is largely coincidental with nuclei. Scale bar = 100 μm in panel A; = 20 μm in panel D, and; = 5 μm in panel G. [file 1471-213X-9-72-S2.PDF]

A

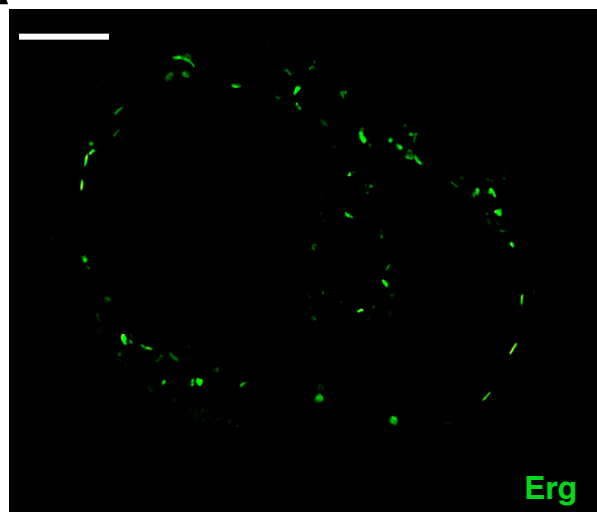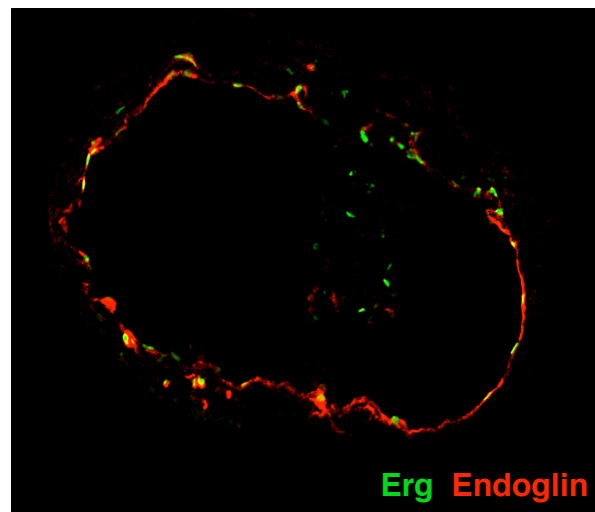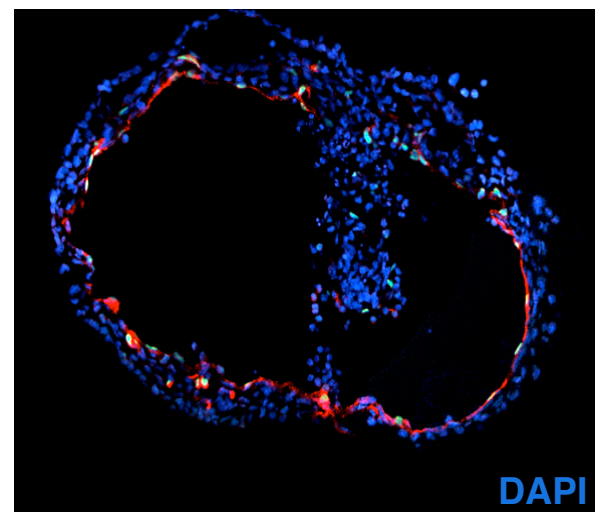

B

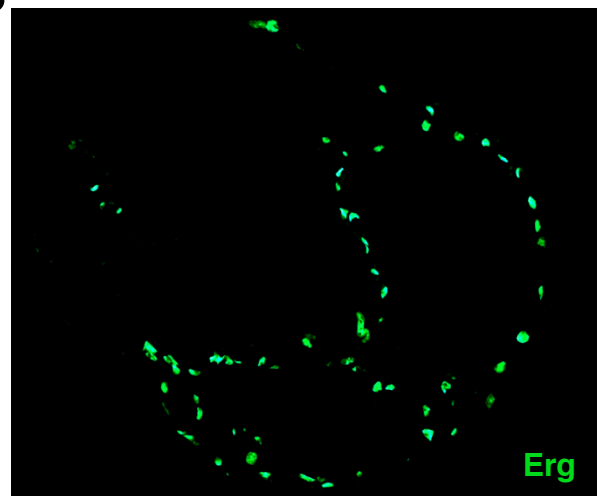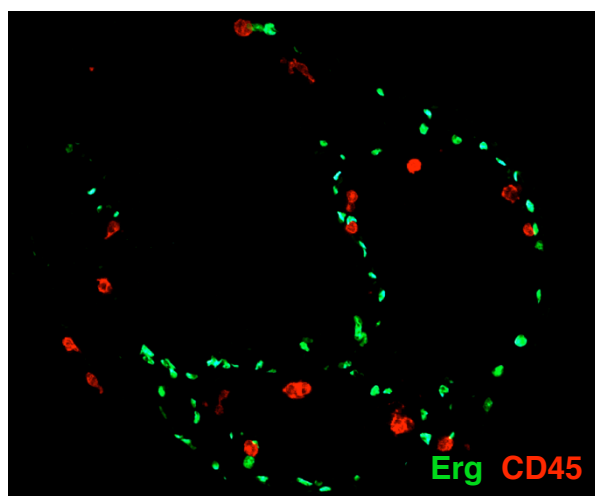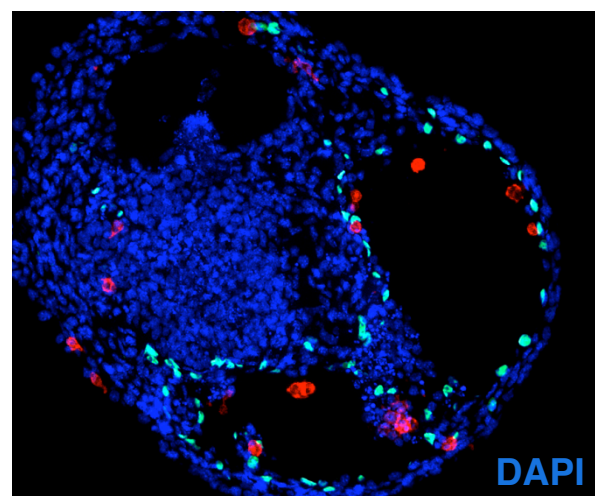

Supplement: Additional file 3 — Fluorescence microscopy evaluation of ERG expression with endoglin and CD45 in day 10 EBs. Panel (A) and (B) show double fluorescence immunostaining of ERG (green) with the endothelial-specific endoglin (red) and hematopoietic cell marker CD45 (red), respectively. Panel (A) demonstrates ERG co-localization with endoglin in the endothelial cells that line the vascular channel walls of the cystic EBs, while in panel (B), CD45 expression is seen in hematopoietic progenitor cells that bud off from the channel's wall and migrate towards the center of the vascular channel lumen. Scale bar = 100 μm. [file 1471-213X-9-72-S3.PDF]

ERG

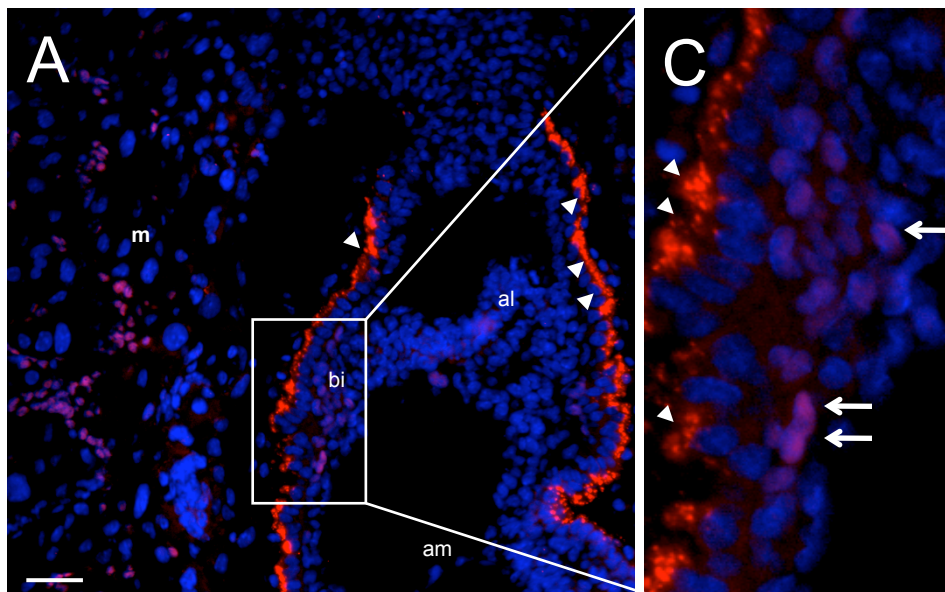

VEGF-R2

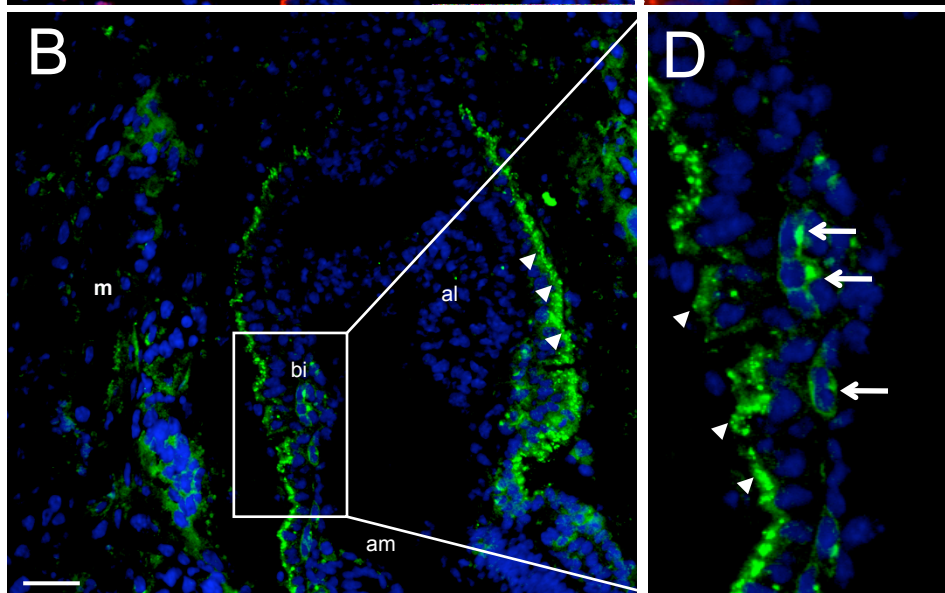

Supplement: Additional file 4 — ERG and VEGF-R2 expression in E7.5 embryo. (A-B)Serial sections of mouse E7.5 embryo showing expression of ERG and VEGF-R2 in a similar subset of cells in the blood islands, respectively. Arrowheads indicate nonspecific staining of the antibodies. (C, D) Higher magnification of (A, B). Arrows point to the cells of the blood islands that express ERG within the nucleus (C) and VEGF-R2 on the surface (D) al, allantois; am, amnion; bi, blood island; m, maternal decidua. Scale bars: 50 μm [file 1471-213X-9-72-S4.PDF]

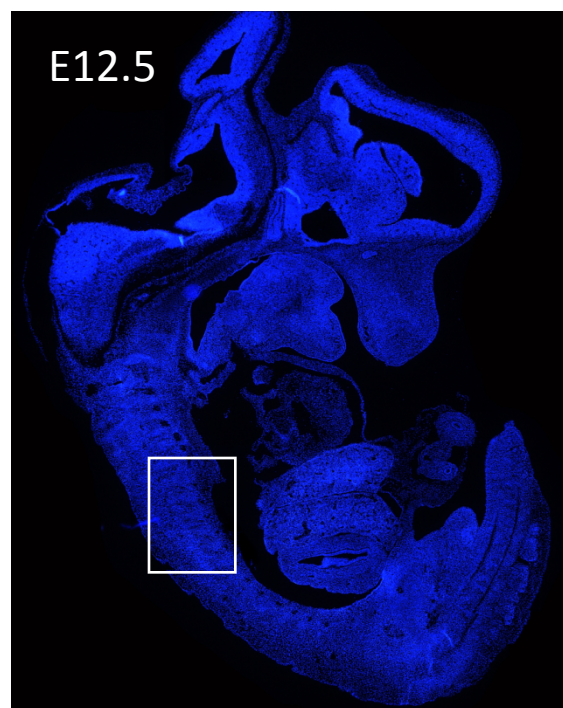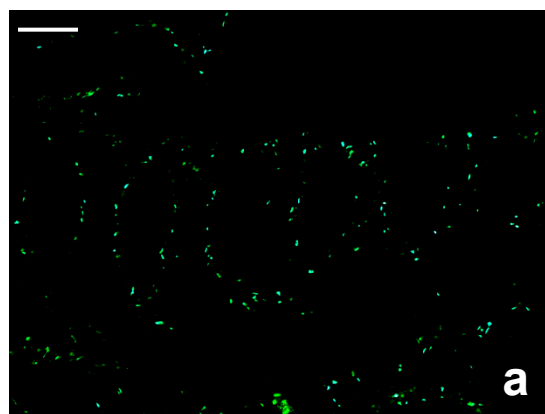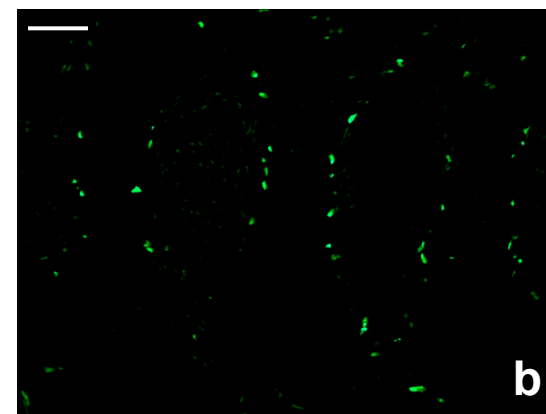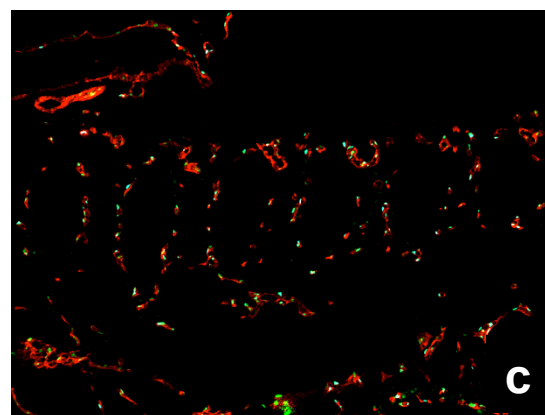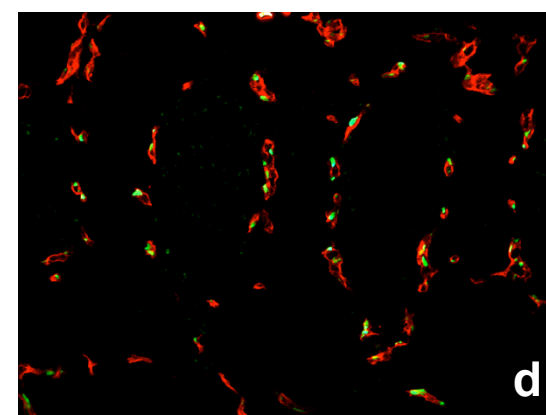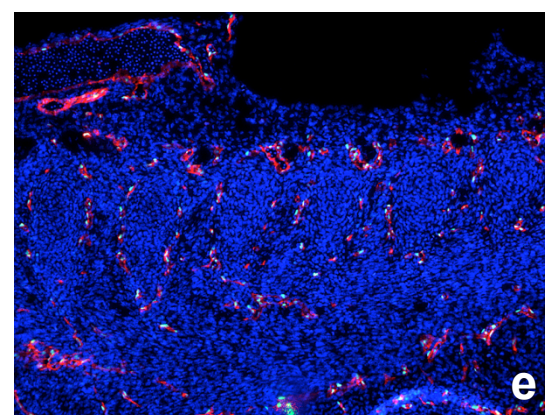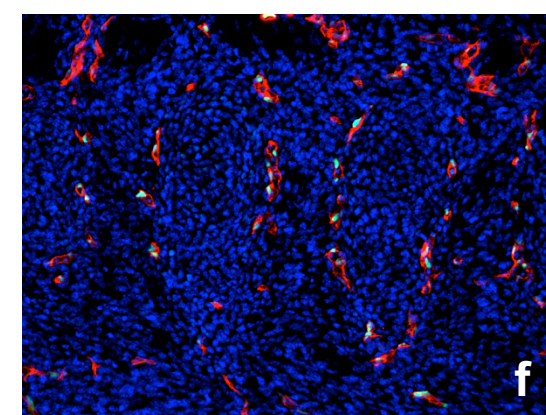

Supplement: Additional file 5 — ERG expression in intersomatic blood vessels in the developing embryo at E12.5. Sagittal sections of frozen mouse embryo at E12.5 (far left image) fluorescence stained with DAPI nuclear stain (blue). The inset box points to the developing somite region in the mouse embryo, which is 20× magnified in the middle column (a, c and e) and 40× magnified in the far right column (b, d and f). Both columns demonstrate ERG co-localization (a, b, green) with the endothelial-specific VE-cadherin (c, d, red) in the developing intersomatic blood vessels. Image (e) and (f) are overlays with DAPI (blue) which outlines the general tissue morphology. Images were obtained with Leica fluorescent microscope. Scale bars: 80 μm (a, c and e) and 30 μm (b, d and f). [file 1471-213X-9-72-S5.PDF]

**A**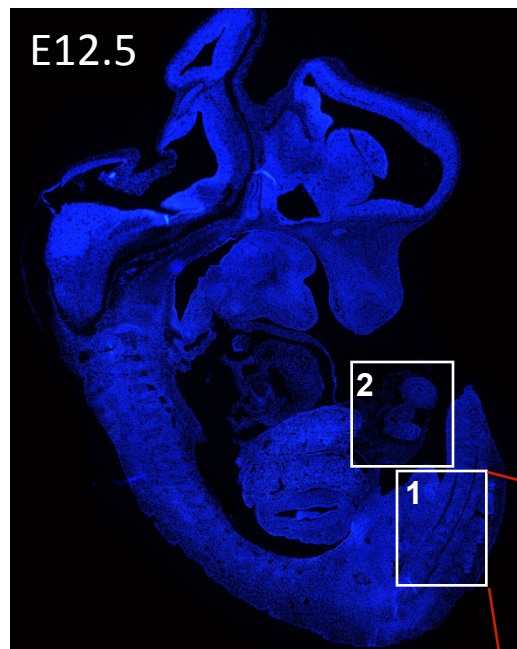**B**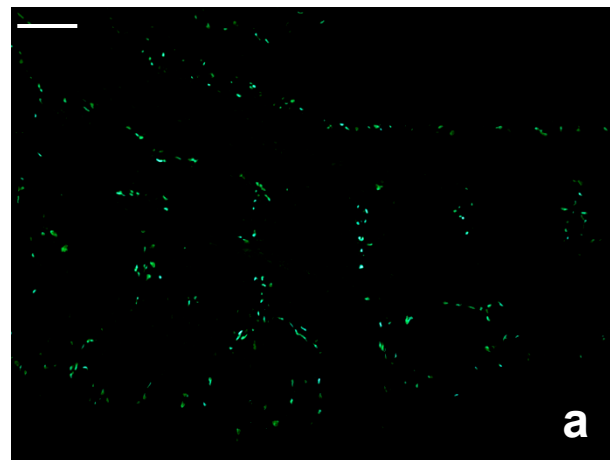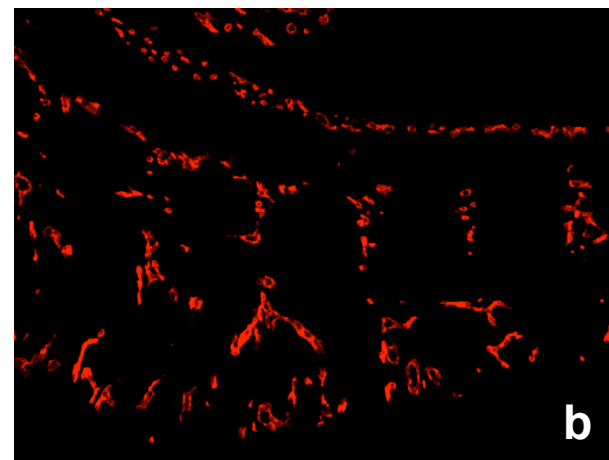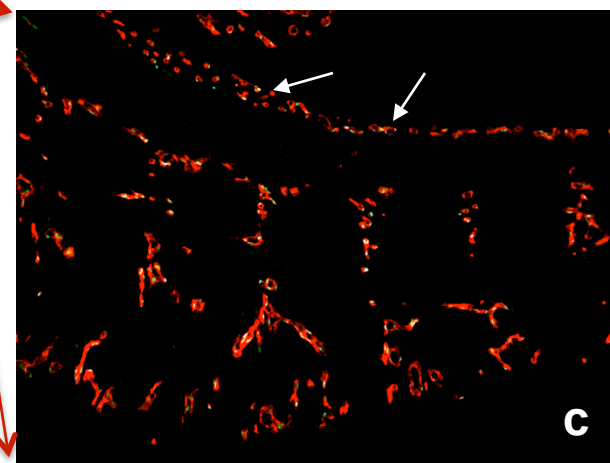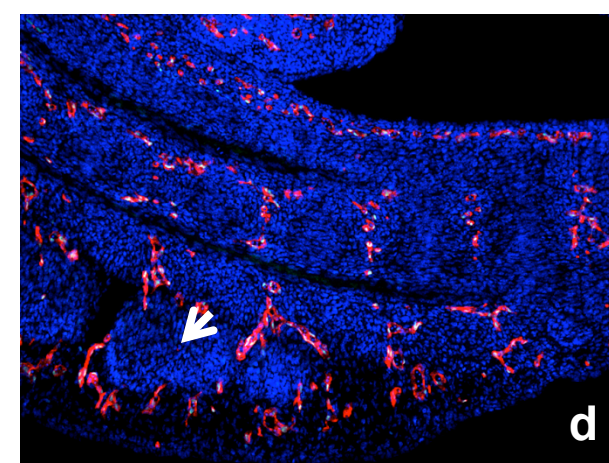

Supplement: Additional file 6 — ERG expression in the aorta-gonado-mesonephros (AGM) region of the developing mouse embryo at E12.5. (A) Sagittal section of a frozen mouse embryo at E12.5, fluorescence stained with DAPI nuclear stain (blue). The inset boxes 1 and 2 outline the AGM and umbilical cord remnant, respectively, in the E12.5 mouse embryo. Panel (B) represents magnification of inset 1 and shows ERG co-expression (a, green) with the endothelial-specific VE-cadherin (b, red) in the developing vasculature of the embryonic AGM region. (c) is a merged image of (a) and (b), while (d) is an overlay with DAPI (blue). The thin arrows in (c) point to the mesonephric blood vessels of the AGM region, while the thick arrow in (d) points to the dorsal (posterior) root ganglion of AGM. Images in panel (A) and (B) were taken with Leica fluorescent microscope at 10× and 20× magnification, respectively. Scale bar = 30 μm. [file 1471-213X-9-72-S6.PDF]

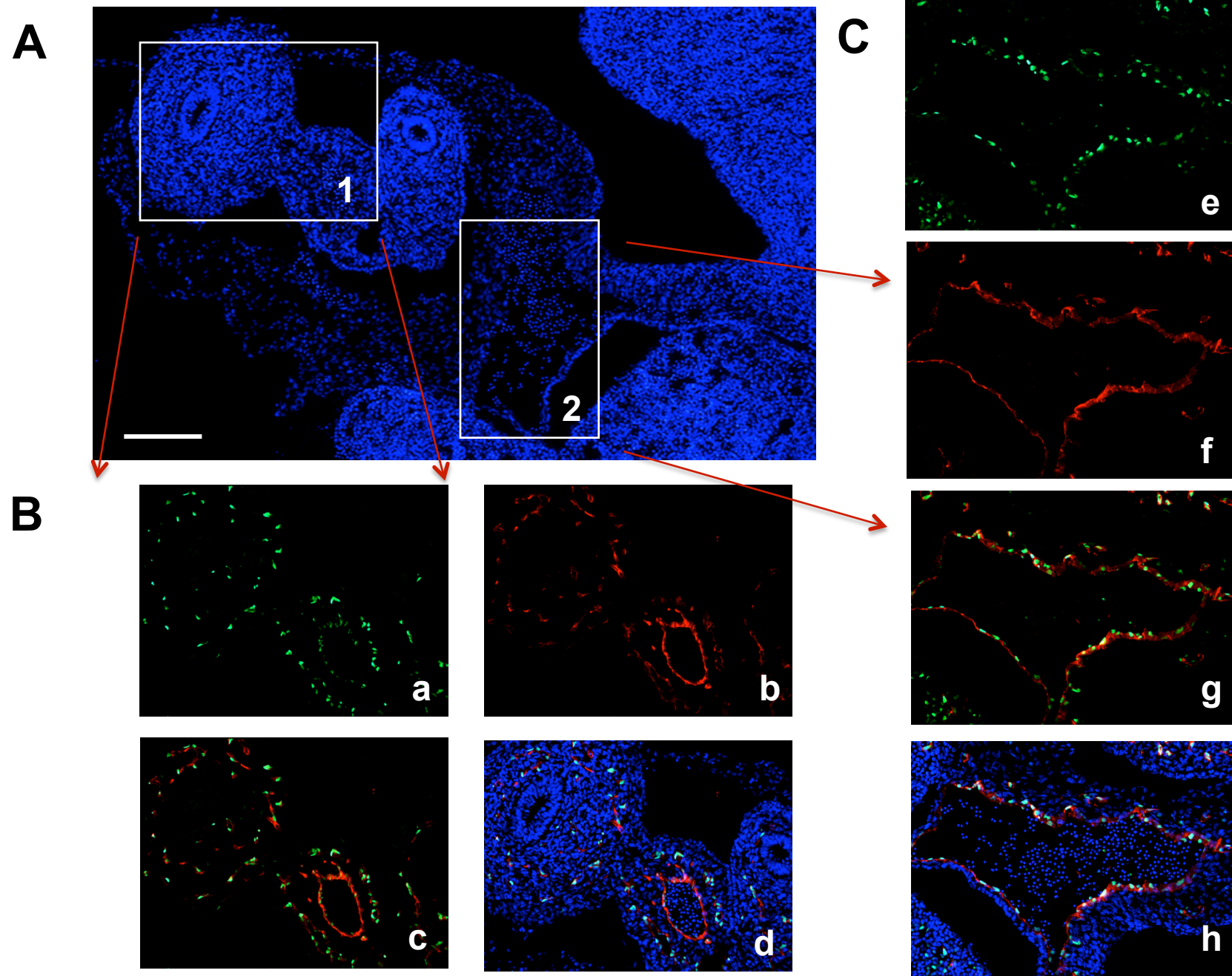

Supplement: Additional file 7 — ERG expression in the umbilical cord of day E12.5 embryo. (A) Magnification of inset box 2 from Additional file 6 showing the DAPI stained remnants of the placenta and umbilical cord vein, respectively. ERG (green, a and e) also co-localizes with VE-cadherin (red, b and f) in the umbilical cord arteries (panel B) and umbilical cord vein (panel C). Scale bar represents 90 μm. [file 1471-213X-9-72-S7.PDF]

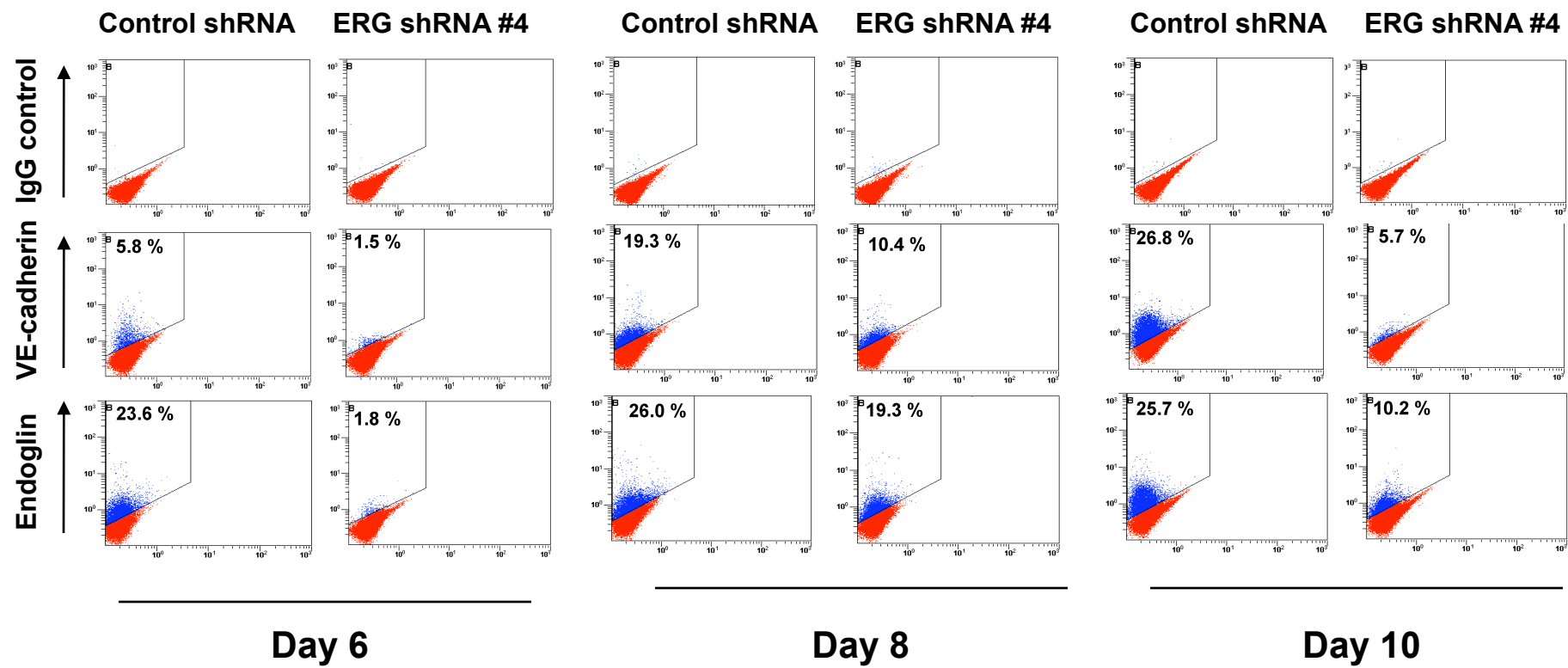

Supplement: Additional file 8 — Effect of ERG knockdown on its direct targets by flow cytometry analysis at different time points. Flow cytometry analysis of the ERG target genes VE-cadherin and endoglin during EB differentiation in ERG shRNA control and shRNA #4 cells. Each flow cytometry diagram is labeled with PE-conjugated antigen-specific Ab that migrates along the y-axis. [file 1471-213X-9-72-S8.PDF]

A

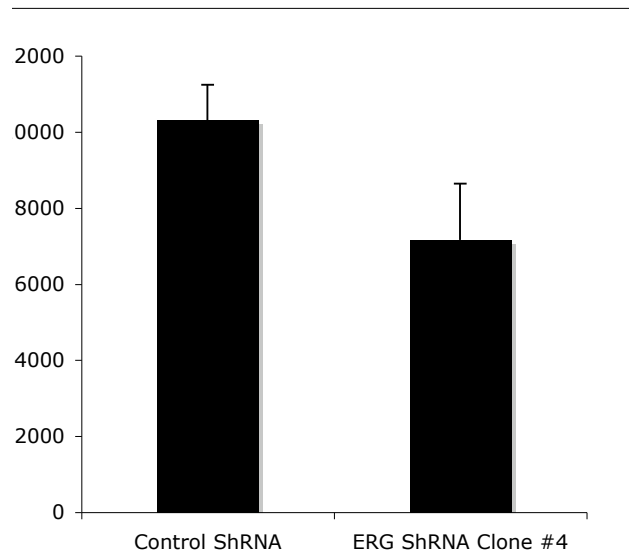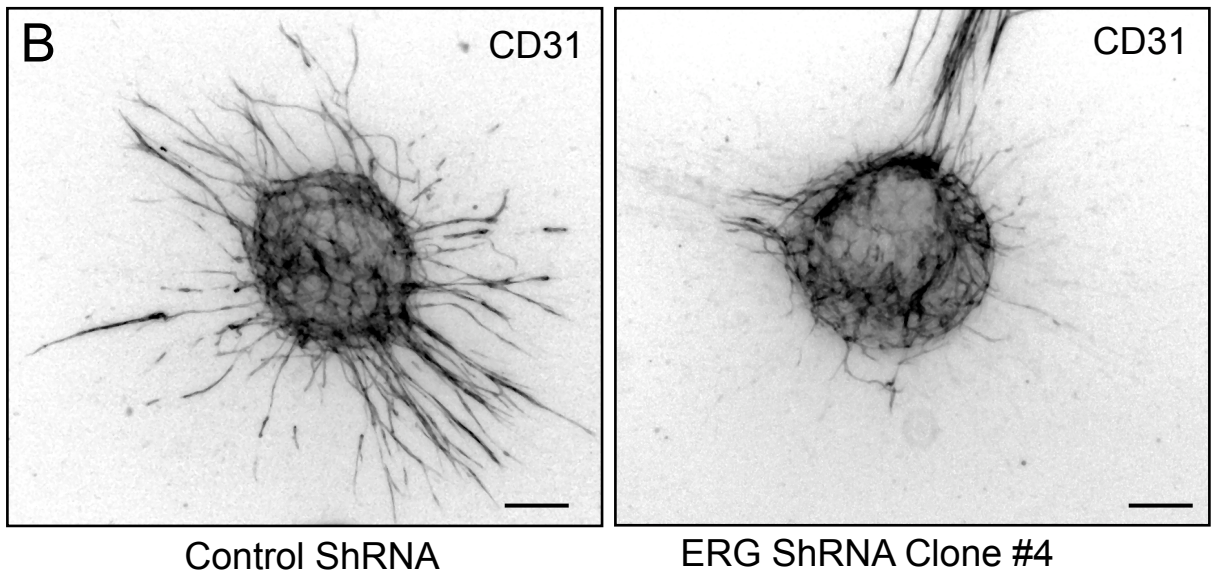

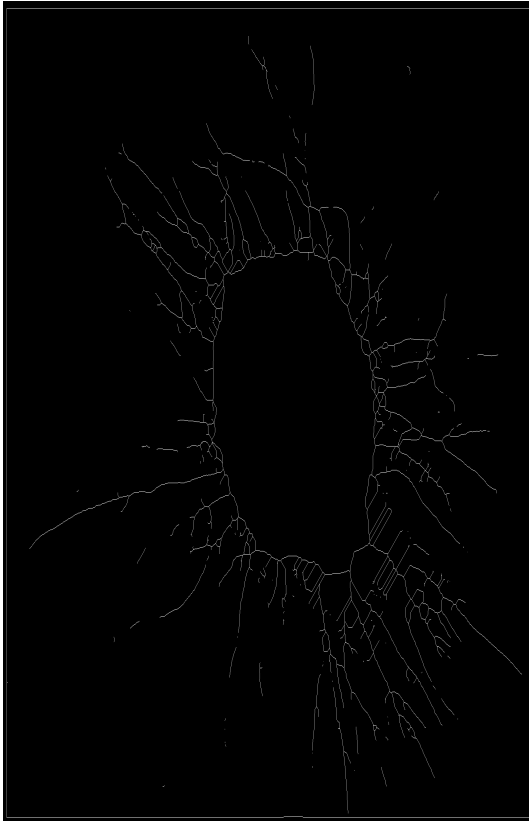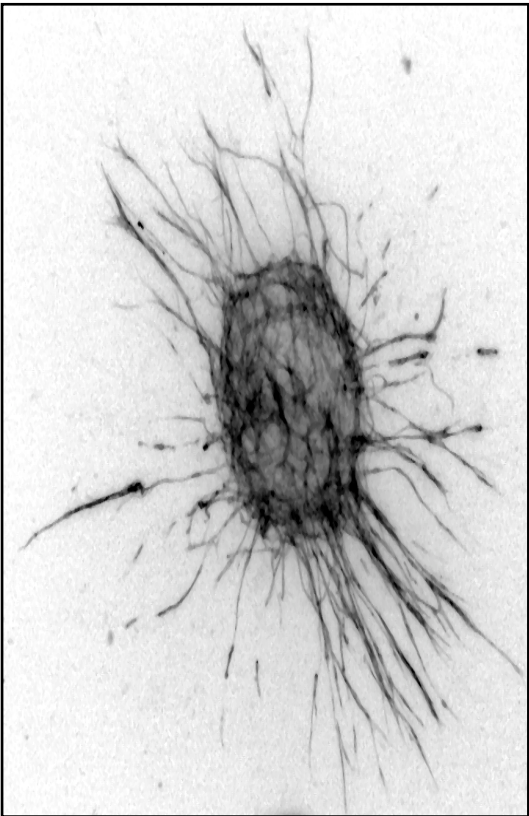

Supplement: Additional file 9 — The effect of ERG knockdown on in-vitro vascular sprout formation on differentiating EB's. (A). Comparison of mean total length of vascular sprouts in control and ERG ShRNA knockdown EB's. Error bars indicate means +/- S.D. (n = 2, with 50-100 EBs per experiment) (B). Representative examples of EBs stained with CD31. Scale bar, 100 μm. [file 1471-213X-9-72-S9.PDF]

**A**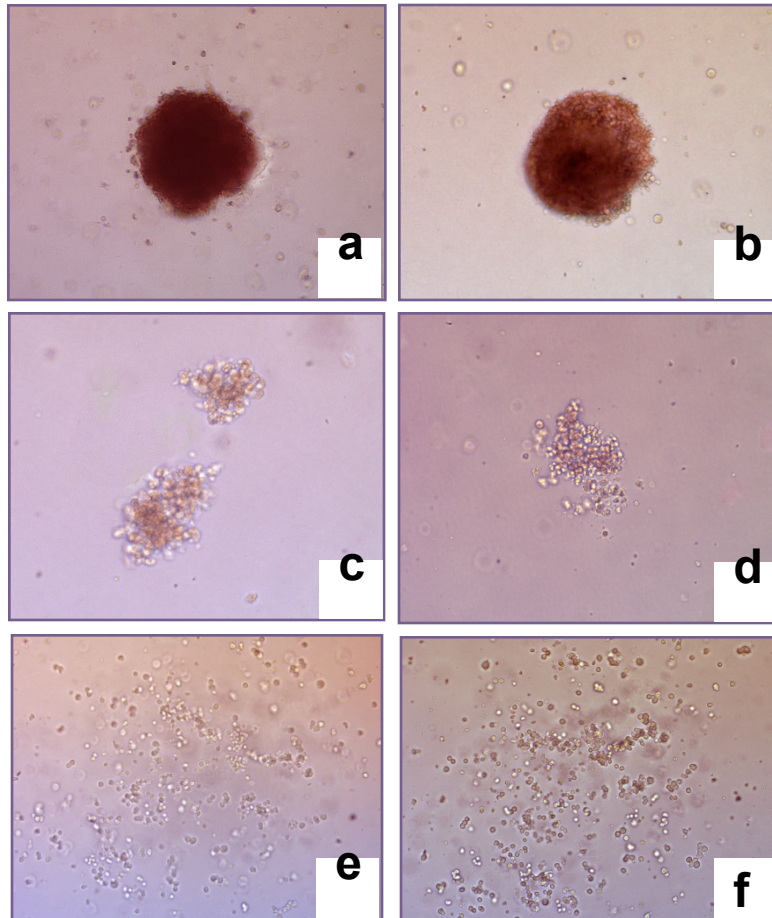**B**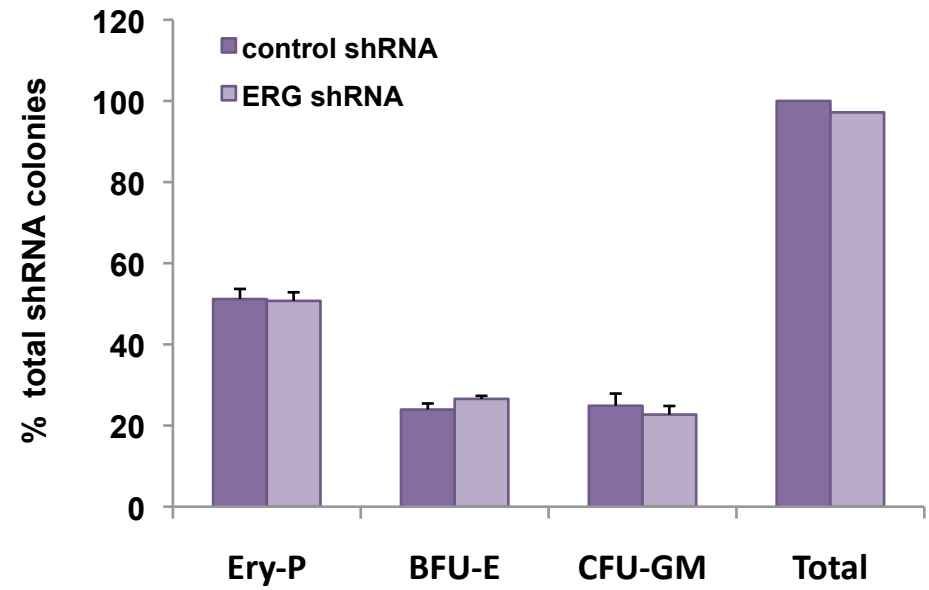**C**

| colonies      | Ery-P     | BFU-E     | CFU-GM    | Total # |
|---------------|-----------|-----------|-----------|---------|
| control shRNA | 54.5±3.54 | 25.5±3.54 | 26.5±2.12 | 106.5   |
| ERG shRNA     | 52.5±3.54 | 28±0.71   | 24±2.12   | 104.5   |

Average # of colonies/plate (n=3)

Supplement: Additional file 10 — Hematopoietic colony forming assay in control and ERG shRNA treated ES cells. (A) Hematopoietic colonies including primitive erythroid colony Ery-P (a and b), blast colony forming unit containing definitive erythroid progenitors BFU-E (c and d), and colony forming units with granulocytes and macrophages CFU-GM (e and f) were formed by both, control shRNA ES cells (a, c, and e) and ERG shRNA treated ES cells (b, d, and f), when grown on methylcellulose base and in presence of hematopoietic growth factors. Images were taken on Nikon light microscope at 10× and 20× magnification. (B) Hematopoietic potential of control and ERG shRNA treated ES cells. The number of individual colonies (Ery-P, BFU-E, and CFU-GM) is expressed as percentage of the total number of colonies for each phenotype (control vs. ERG shRNA ES cells). (C) Values used in the table represent average number of counted colonies from three individual plates (n = 3). Error bars indicate means +/- S.D. [file 1471-213X-9-72-S10.PDF]

**A**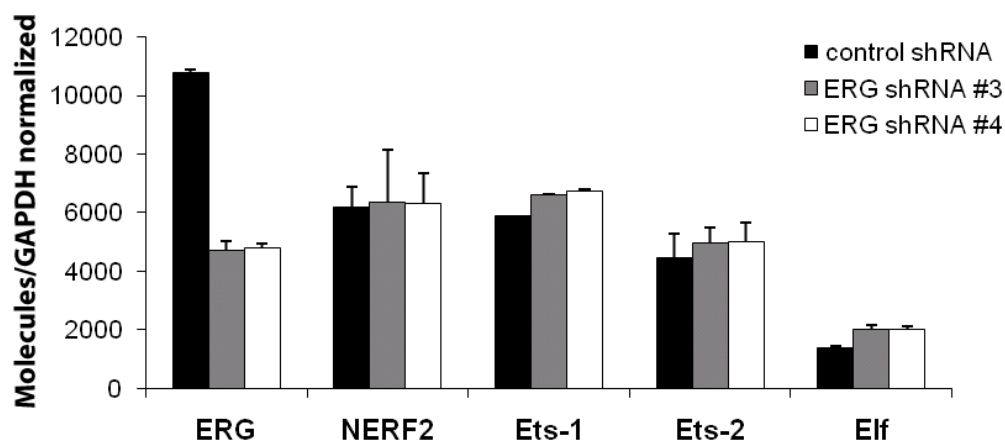**B**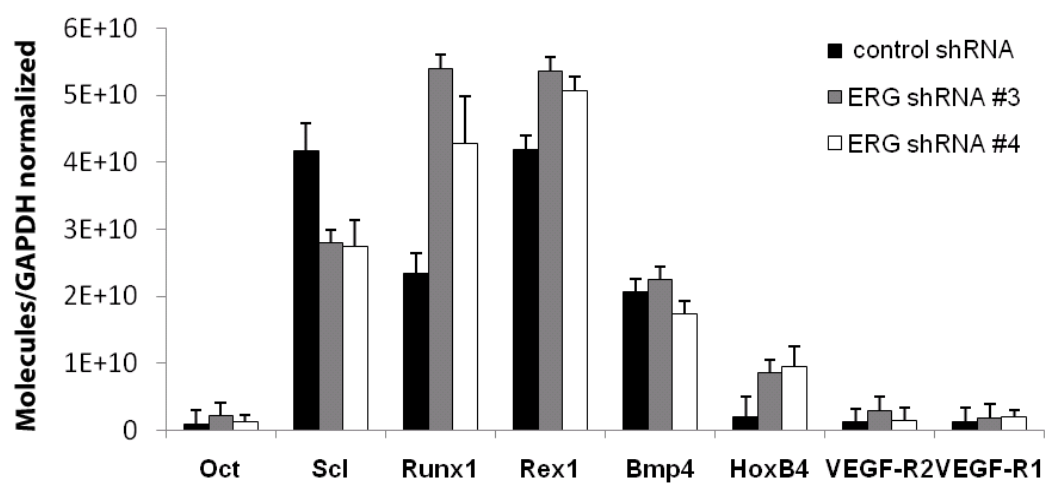**C**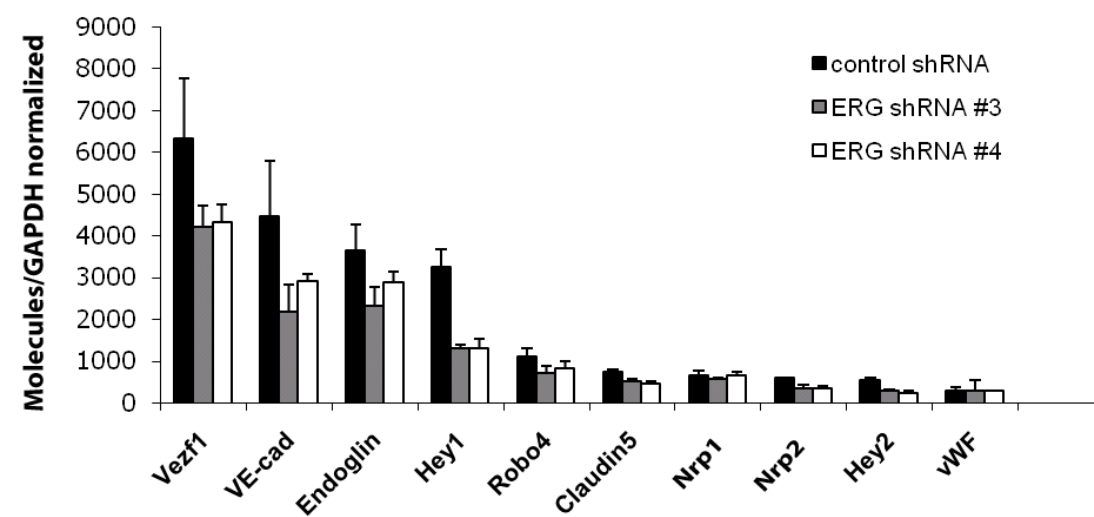

Supplement: Additional file 12 — Real-time RT-PCR analysis of control and ERG shRNA treated EBs at day 4.5. (A) Expression of ETS family of transcription factors in control shRNA and ERG shRNA treated EBs at day 4.5. (B) QRT-PCR evaluation of expression of mesodermal and hematopoietic markers in control and ERG shRNA treated EBs at day 4.5 of differentiation. (C) Analysis of expression of different endothelial-cell specific markers in control and ERG shRNA treated EBs at day 4.5. In all three experiments differential expression of cell-lineage specific markers in control EBs was compared with EBs treated with two different ERG shRNAs: sequence #3 and #4. For each molecular marker tested n = 3 and the error bars indicate means +/- S.D. [file 1471-213X-9-72-S12.PDF]

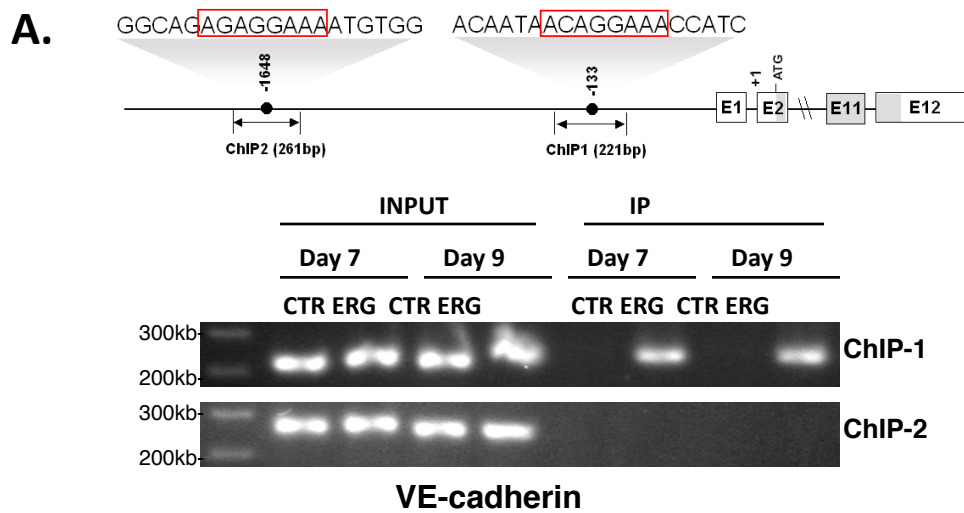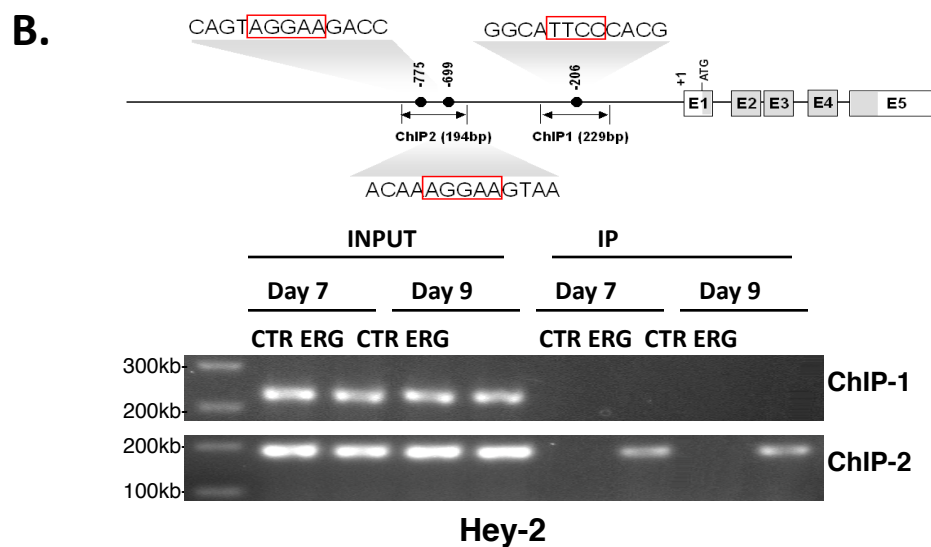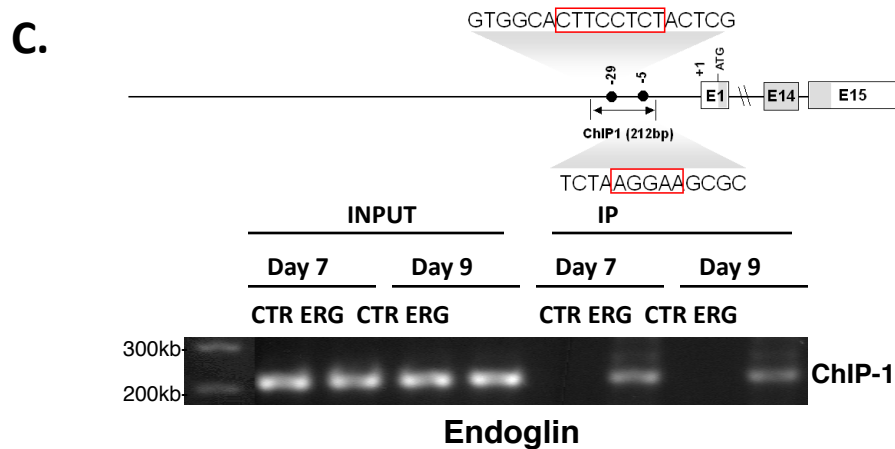

Supplement: Additional file 13 — Downstream targets of ERG. (A, B, C) Top: Schematic diagram of ERG binding sites in VE-cadherin, Hey-2, or Endoglin promoter, respectively. The 1.5 kb upstream promoter region of each gene was analyzed in search for the potential ERG binding sites indicated with red boxes. The bidirectional arrows marked the target regions for ChIP assays (ChIP1 and/or ChIP2). (A, B, C) Bottom: ChIP assay using HUVEC. An ERG polyclonal antibody was used for precipitation. PCR analysis of the input, in the absence of ERG antibody (CTR), and in the presence of ERG antibody (ERG) after immunoprecipitation (IP) using primers corresponding to indicated ERG putative binding sites (ChIP1 and/or ChIP2) of the each gene promoter. Molecular weight markers are shown on the left. [file 1471-213X-9-72-S13.PDF]
