# Supplementary material for: ERG is required for the differentiation of embryonic stem cells along the endothelial lineage
Source: BMC Dev Biol. 2009 Dec 23;9:72. doi: 10.1186/1471-213X-9-72 (PMC2803788; doi:10.1186/1471-213X-9-72)
Supplement: Additional file 11 — List of differentially expressed genes in ERG shRNA versus control ES cells. Selected list of genes that are significantly changed after 3 days or 4 of ES cell differentiation in the control versus ERG shRNA treated cells. The Fold change (FC) and Lower Bound of Fold change (LCB) of significantly changing genes is shown in BOLD. [file 1471-213X-9-72-S11.PDF]

Gene	Name	3 days		4 days	
		FC	LCB	FC	LCB
**Sox18**	SRY-box containing gene 18	**-6.43**	**-5.41**	**-3.52**	**-3.0**
**Esam**	endothelial cell-selective adhesion molecule	**-3.55**	**-2.92**	**-3.26**	**-2.8**
**Cdh5**	cadherin 5	**-5.32**	**-3.84**	**-2.7**	**-2.3**
**Edg1**	endothelial differentiation sphingolipid G-protein-coupled receptor 1	**-6.21**	**-4.03**	**-2.11**	**-1.8**
**Flt4**	FMS-like tyrosine kinase 4	**-3.95**	**-3.65**	**-1.89**	**-1.7**
**Hhex**	hematopoietically expressed homeobox	**-4.59**	**-3.91**	**-1.84**	**-1.7**
**Tcfec**	transcription factor EC	**-3.12**	**-2.48**	**-1.98**	**-1.7**
**Hey1**	hairy/enhancer-of-split related with YRPW motif 1	**-4.45**	**-4.21**	**-2.02**	**-1.6**
**Nrp1**	neuropilin	**-2.86**	**-2.26**	**-1.81**	**-1.6**
**Sox7**	SRY-box containing gene 7	**-5.86**	**-4**	**-1.69**	**-1.6**
**Arhj**	ras homolog gene family, member J	**-6.64**	**-4.17**	**-1.44**	**-1.3**
**Erg**	avian erythroblastosis virus E-26 (v-ets) oncogene related	**-5.49**	**-4.34**	**-1.55**	**-1.2**
**Tek**	endothelial-specific receptor tyrosine kinase	**-2.38**	**-2.06**	**-1.3**	**-1.2**
**Idb4**	inhibitor of DNA binding 4	**-4.86**	**-4.19**	**-1.29**	**-1.2**
**Sox17**	SRY-box containing gene 17	**-5.16**	**-3.98**	**-1.63**	**-1.2**
**Nfatc1**	nuclear factor of activated T-cells, cytoplasmic 1	**-3.83**	**-3.11**	-1.67	-1.1
**Bmp2**	bone morphogenetic protein 2	**-4.17**	**-2.89**	-1.25	-1.1
**Cdh5**	cadherin 5	**-8.54**	**-6.35**	-1.24	-1.1
**Gata2**	GATA binding protein 2	**-4.92**	**-3.59**	-1.23	-1.0
**Sox9**	SRY-box containing gene 9	**-7.44**	**-5**	-1.04	-0.9
**Bmp6**	bone morphogenetic protein 6	**-3.51**	**-2.86**	-1.28	-0.8
**Flt1**	FMS-like tyrosine kinase 1	**-4.02**	**-3.39**	-1.12	-0.8
**Bmper**	BMP-binding endothelial regulator	**-3.05**	**-2.48**	-1.02	-0.8
**Nrp2**	neuropilin 2	**-3.42**	**-2.9**	1.03	0.9
**Hoxd1**	homeo box D1	**-3.63**	**-2.62**	1.34	1.2
