# Supplementary material for: ERG is required for the differentiation of embryonic stem cells along the endothelial lineage
Source: BMC Dev Biol. 2009 Dec 23;9:72. doi: 10.1186/1471-213X-9-72 (PMC2803788; doi:10.1186/1471-213X-9-72)
Supplement: Additional file 14 — Supplemental Methods. Supplemental methods not described in the main document. [file 1471-213X-9-72-S14.PDF]

SUPPLEMENTAL METHODS AND FIGURE LEGENDS:

Immunohistochemistry of ERG expression in embryoid bodies and mouse embryos: EBs from different time points (day 3.5 to 10.5) were washed with PBS and embedded in OCT freezing compound (Miles Scientific, Cambridge, ML) after fixation in 4% paraformaldehyde for 1 hour at 4°C and cryoprotection in 20% (wt/vol) sucrose overnight. Frozen sections (8-10 μm thick) were mounted onto gelatin-coated glass slides, air-dried for 30 min, permeabilized for 3 minutes with 0.5% Triton X-100, and washed two times with PBS. Slides were then processed for immunofluorescence microscopy using primary rabbit polyclonal ERG antibody (1:200, Santa Cruz) and rat monoclonal VE-cadherin, CD41, CD45 and endoglin antibodies (1:100, Pharmingen). Secondary antibodies used were green anti-rabbit Alexa-488 and red anti-rat Alexa-594 from Molecular Probes. Mouse embryos were harvested at different stages of embryogenesis (every day from E7.5 to E14.5). The embryos were washed twice in PBS, fixed in 4% paraformaldehyde for 16 hours at 4°C, after which they were perfused with 30% sucrose in PBS (w/v) and frozen in OCT media for cryosectioning. Serial transverse and sagittal sections, 6μm thick, were obtained and co-immunostained with rabbit anti-ERG and rat anti-VE-cadherin antibody for 1 hour at room temperature, followed by 45 min incubation with the same secondary antibodies. Images were taken by using Nikon digital camera operated with the OpenLab software.

Confocal Microscopy Image Acquisition and Processing: Imaging was conducted on a Zeiss LSM 510 laser-scanning confocal microscope with a 40x oil-immersion objective.

Digital images were then exported to Adobe Photoshop software for processing and final image preparation.

Lentiviral shRNA for knockdown studies in ES cells: Undifferentiated ES cells were plated one day before infection onto 24 well plates. On the day of infection, the medium was replaced with 250 μl OptiMEM. Lentivirus encoding the shRNA directed against particular gene targets of interest (Sigma) and polybrene (8 μg/ml; Sigma, Cat.# H9268) was added to the ES cells and incubated for 4 hours, after which 1 ml of ES media was added to the infected cells. The cells were then incubated for 48 hours before puromycin selection of infected cells was performed. For puromycin selection, 1 μg/ml puromycin (Sigma) was added to the culture media. Stable clones were selected and expanded.

Sprouting Assay: The assay was performed as reported by Perlingeiro et al [1]. Erg ShRNA control and clone #4 were differentiated into EB's at 2 x 10^3 cells per 35-mm culture dish (# 27100, Stem Cell Technologies) in ES-Cult M120 methyl-cellulose based medium (#M3120, Stem Cell Technologies) supplemented with 15% FBS (#SO1520ESL, USA Scientific), 10mg/ml recombinant human insulin (19278, Sigma) , 50ng/ml human VEGF (#02628, Stem Cell Technologies), 100ng/ml human FGF-b (#02634, Stem Cell Technologies), 10ng/ml hIL6 (#PHC0065, Invitrogen), 2U/ml hEPO, (#PHC2054, Invitrogen), and 45mM MTG. After 11 days of EB differentiation, intact EB's were washed with IMDM medium and cultured at ~100 EBs per 35-mm Petri dish in ES-Cult Endothelial basal medium and collagen 1 matrix (#05810, Stem Cell Technologies) supplemented

with 50ng/ml hVEGF, 100ng/ml hFGF-b, 10ng/ml hIL-6 and 2U/ml hEPO. After 2 days at 37°C and 5% CO_2, the EBs were fixed with 4% PFA for 30 min, and then permeabilzed with 0.2% Triton X-100 in TBS. Blocking against unspecific binding was performed for 1h with 10% FCS / 0.01% TBST. The primary rat anti-mouse CD31 antibody (BD) incubation was performed at 4°C overnight at a dilution of 1:100. The following day, the EBs were washed for 30 mins in TBS and incubated for 1h with goat anti-rat Alexa 488 (Invitrogen) secondary antibody. The EBs were then washed in TBS for 30 min, transferred to glass slides, and mounted in ProlongGold antifade reagent (Invitrogen). Computer-assisted morphometric analysis of vascular sprouting was performed as reported by Feraud et al., 2001 [2].

Flow cytometry and Fluorescence-activated cell sorting (FACS). EBs were collected at different time points of differentiation, as described earlier. After being washed twice in PBS (without Ca^{2+} and Mg^{2+}), they were broken down into a single cell suspension by first incubating them in a non-enzymatic cell dissociation solution (Sigma) for 20-30 min at 37°C, and then pipetting and filtering the cells through a 100 μm cell-strainer. The cells were then used for single-color labeling with anti-ERG (Santa Cruz) followed by secondary antibody incubation with FITC (Jackson Research Laboratories). For double labeling with anti-ERG,-FITC, the cells were further incubated for 30 minutes with each of the following PE-conjugated antibodies CD41, TER119 (Pharmingen) and unconjugated VE-Cadherin (Pharmingen). For the time course analysis, in addition to the above antibodies, Tie2-PE (eBioscience), Flt1-PE (R&D Systems) and Endoglin (Pharmingen)

were used for single color labeling. Primary antibody isotypes were used as controls for non-specific background staining. All antibody incubations were done at 4°C for 30 min. The labeled cells were washed with PBS and fixed in 1% paraformaldehyde for preservation. Flow cytometry runs and FACS sorting were performed on the FX5000 Flow Cytometer/FACS sorter at the Beth Israel Medical Center Flow Cytometry Facility, using the CXP analysis software.

Quantitative RT-PCR: QRT-PCR was used to compare the temporal expression pattern of ERG with the other ETS factors and vascular specific genes at various stages of ES differentiation. Cells from whole EBs and EB-derived sorted ES cells were collected at various stages of differentiation and total RNA was isolated using the Qiagen RNAeasy extraction kit. Single stranded cDNA was synthesized from total RNA with oligo(dT) 12-18 priming, using the Moloney murine leukemia virus reverse transcriptase (Gibco).

Hematopoietic Blast colony-forming cell (BL-CFC) assay in methylcellulose: Hematopoietic potential of the control shRNA, and ERG shRNA CCE ES cells was tested by performing the BL-CFC assay using the ES-Cult Starter Kit with Cytokines (StemCell Technologies, Cat.# 03161). The methodology was carried out as per instructions manual. In brief, ES cells were harvested using 0.25% trypsin-EDTA and resuspended in primary differentiation media (PDM) containing Iscov's Modified Dulbecco's Media (IMDM), 1% methylcellulose (Cat.# M3120), 15% FBS, 2 mM L-glutamine, 150 mM MTG (Sigma) and 40 ng/ml murine stem cell factor (mSCF), ensuring that a single cell

suspension is achieved with density of 2-5x10^3 cells/mL. To obtain 50 to 100 EBs per 35 mm Petri dish, ES cells were plated in the appropriate range between 200 to 500 cells per dish. EBs were visible by day 2-3 of differentiation and were grown in culture for 8 days.

Microarray analysis: Microarray analysis was conducted via the Genomics Center at the Beth Israel Deaconess Medical Center, according to previously described protocols for total RNA extraction and purification, cDNA synthesis, *in vitro* transcription reaction for production of biotin-labeled cRNA, hybridization of cRNA with mouse genome 430 2.0 Affymetrix gene chips, and scanning of image output files. The quality of scanned arrays images were determined on the basis of background values, percent present calls, scaling factors, and 3'-5' ratio of β-actin and GAPDH using the BioConductor R packages. Scanned array images were analyzed by dChip, as it is more robust than MAS5.0 and RMA in signal calculation. The raw probe level data was normalized using smoothing-spline invariant set method. The signal value for each transcript was summarized using PM-only based signal modeling algorithm described in dchip. The PM only based modeling based algorithm yields less number of false positives as compared to the PM-MM model. In this way, the signal value corresponds to the absolute level of expression of a transcript. These normalized and modeled signal values for each transcript were used for further high-level bioinformatics analysis. During the calculation of model based expression signal values, array and probe outliers are interrogated and images spike are treated as signal outliers. The outlier detection was carried out using dchip outlier detection algorithm. A chip is considered as an outlier if the probe, single or array outlier

percentage exceeds a default threshold of 5%.

When comparing two groups of samples to identify genes enriched in a given phenotype, if 90% lower confidence bound (LCB) of the fold change (FC) between the two groups was above 1.5, the corresponding gene was considered to be differentially expressed LCB is a stringent estimate of FC and has been shown to be the better ranking statistic [3] [4, 5]. It has been suggested that a criterion of selecting genes that have a LCB above 2.0 most likely corresponds to genes with an "actual" fold change of at least 3 in gene expression.

<u>Chromatin immunoprecipitation (ChIP) analysis of selected gene targets:</u> ChIP analysis was performed using the ChIP Assay Kit (Upstate Biotechnology, Waltham, MA) according to the manufacturer's protocols. PCR was performed using primers: 1) mVE-cad-ChIP1: sense, 5'-GGCTGAATGCTCTGGCAGAAGCT-3' and antisense, 5'-CACTTCCCCTCGGGATGGTT-3': 2) mVE-cad-ChIP2: sense, 5'-AGTGCTACCCTGGCAGACGT-3' and antisense, 5'- TGGGGCTGTTCTCAGCTCTG-3'. 3) mHey2-ChIP1: sense, 5'-CTTGCAGGTGATGGCCCAAAACC-3' and antisense, 5'-TCCCTGGGACGGTGTGGCTT-3'. 4) mHey2-ChIP2: sense, 5'-TCCCATCAGTAGGAAGACCACTCA-3' and antisense, 5'-GCCTCTAGGTTGTGGTCAGA-3'. 5) mEng: sense, 5'-AAGGTTGCCAGATGTGTCCTCA-3' and antisense, 5'-GAGGCAGGAAATGCGCTTCC-3'.

Additional File Legends

Additional file 1. Flow Cytometry of ERG and Ter119 during ES cell differentiation. Expression of ERG (FITC labeled) and Ter119 (PE-labeled) was analyzed using the flow-cytometry technique in day 8.5, 10.5 and 12.5 EBs. Flow-cytometry diagrams show expression of the two markers on clearly two distinct cell populations (Ter199 population migrating along the y-axis, while the ERG population shifts along the x-axis).

Additional file 2. Confocal expression of ERG. EB sections were stained for ERG (green), VE-cadherin (red), and nuclei (blue) and visualized by laser-scanning confocal microscopy. A-C. ERG **(A)**, VE-cadherin **(B)** and merge of all three stains is depicted in **(C)**. **(D)** Depicts a subregion as indicated in panel C. **(E-G)** These panels depict a subregion of panel D as indicated, and show ERG **(E)** and nuclei **(G)** alone or merged **(F)**. Note that ERG staining is largely coincidental with nuclei. Scale bar = 100 μm in panel A; = 20 μm in panel D, and; = 5 μm in panel G.

Additional file 3. Fluorescence microscopy evaluation of ERG expression with endoglin and CD45 in day 10 EBs. Panel **(A)** and **(B)** show double fluorescence immunostaining of ERG (green) with the endothelial-specific endoglin (red) and hematopoietic cell marker CD45 (red), respectively. Panel (A) demonstrates ERG co-localization with endoglin in the endothelial cells that line the vascular channel walls of the cystic EBs, while in panel (B), CD45 expression is seen in hematopoietic progenitor cells

that bud off from the channel's wall and migrate towards the center of the vascular channel lumen. Scale bar = 100 μm.

Additional file 4. ERG expression in intersomatic blood vessels in the developing embryo at E12.5. Sagittal sections of frozen mouse embryo at E12.5 (far left image) fluorescence stained with DAPI nuclear stain (blue). The inset box points to the developing somite region in the mouse embryo, which is 20x magnified in the middle column (a, c and e) and 40x magnified in the far right column (b, d and f). Both columns demonstrate ERG co-localization (a, b, green) with the endothelial-specific VE-cadherin (c, d, red) in the developing intersomatic blood vessels. Image (e) and (f) are overlays with DAPI (blue) which outlines the general tissue morphology. Images were obtained with Leica fluorescent microscope. Scale bars: 80 μm (a, c and e) and 30 μm (b, d and f).

Additional file 5. ERG expression in the aorta-gonado-mesonephros (AGM) region of the developing mouse embryo at E12.5. (A) Sagittal section of a frozen mouse embryo at E12.5, fluorescence stained with DAPI nuclear stain (blue). The inset boxes 1 and 2 outline the AGM and umbilical cord remnant, respectively, in the E12.5 mouse embryo. Panel **(B)** represents magnification of inset 1 and shows ERG co-expression (a, green) with the endothelial-specific VE-cadherin (b, red) in the developing vasculature of the embryonic AGM region. (c) is a merged image of (a) and (b), while (d) is an overlay with DAPI (blue). The thin arrows in (c) point to the mesonephric blood vessels of the AGM region, while the thick arrow in (d) points to the dorsal (posterior) root ganglion of

AGM. Images in panel (A) and (B) were taken with Leica fluorescent microscope at 10x and 20x magnification, respectively. Scale bar = 30 μm.

Additional file 6. ERG expression in the umbilical cord of day E12.5 embryo. (A) Magnification of inset box 2 from Additional file 5 showing the DAPI stained remnants of the placenta and umbilical cord vein, respectively. ERG (green, a and e) also co-localizes with VE-cadherin (red, b and f) in the umbilical cord arteries (panel **B**) and umbilical cord vein (panel **C**). Scale bar represents 90 μm.

Additional file 7. ERG and VEGF-R2 expression in E7.5 embryo. (**A-B**)Serial sections of mouse E7.5 embryo showing expression of ERG and VEGF-R2 in a similar subset of cells in the blood islands, respectively. Arrowheads indicate nonspecific staining of the antibodies. (C, D) Higher magnification of (A, B). Arrows point to the cells of the blood islands that express ERG within the nucleus (C) and VEGF-R2 on the surface (D) al, allantois; am, amnion; bi, blood island; m, maternal decidua. Scale bars: 50μm

Additional file 8. Effect of ERG knockdown on its direct targets by flow cytometry analysis at different time points. Flow cytometry analysis of the ERG target genes VE-cadherin and endoglin during EB differentiation in ERG shRNA control and shRNA #4 cells. Each flow cytometry diagram is labeled with PE-conjugated antigen-specific Ab that migrates along the y-axis.

Additional file 9. The effect of ERG knockdown on in-vitro vascular sprout formation on differentiating EB's. (**A**). Comparison of mean total length of vascular sprouts in control and ERG ShRNA knockdown EB's. Error bars indicate means +/- S.D. (n=2, with 50-100 EBs per experiment) (**B**). Representative examples of EBs stained with CD31. Scale bar, 100μm.

Additional file 10. Hematopoietic colony forming assay in control and ERG shRNA treated ES cells. (**A**) Hematopoietic colonies including primitive erythroid colony Ery-P (a and b), blast colony forming unit containing definitive erythroid progenitors BFU-E (c and d), and colony forming units with granulocytes and macrophages CFU-GM (e and f) were formed by both, control shRNA ES cells (a, c, and e) and ERG shRNA treated ES cells (b, d, and f), when grown on methylcellulose base and in presence of hematopoietic growth factors. Images were taken on Nikon light microscope at 10x and 20x magnification. (**B**) Hematopoietic potential of control and ERG shRNA treated ES cells. The number of individual colonies (Ery-P, BFU-E, and CFU-GM) is expressed as percentage of the total number of colonies for each phenotype (control vs. ERG shRNA ES cells). (**C**) Values used in the table represent average number of counted colonies from three individual plates (n=3). Error bars indicate means +/- S.D.

Additional file 11. Real-time RT-PCR analysis of control and ERG shRNA treated EBs at day 4.5. (**A**) Expression of ETS family of transcription factors in control shRNA

and ERG shRNA treated EBs at day 4.5. (**B**) QRT-PCR evaluation of expression of mesodermal and hematopoietic markers in control and ERG shRNA treated EBs at day 4.5 of differentiation. (**C**) Analysis of expression of different endothelial-cell specific markers in control and ERG shRNA treated EBs at day 4.5. In all three experiments differential expression of cell-lineage specific markers in control EBs was compared with EBs treated with two different ERG shRNAs: sequence #3 and #4. For each molecular marker tested n=3 and the error bars indicate means +/- S.D.

Additional file 12. Downstream targets of ERG. (**A, B, C**) Top: Schematic diagram of ERG binding sites in VE-cadherin, Hey-2, or Endoglin promoter, respectively. The 1.5 kb upstream promoter region of each gene was analyzed in search for the potential ERG binding sites indicated with red boxes. The bidirectional arrows marked the target regions for ChIP assays (ChIP1 and/or ChIP2). (A,B,C) Bottom: ChIP assay using HUVEC. An ERG polyclonal antibody was used for precipitation. PCR analysis of the input, in the absence of ERG antibody (CTR), and in the presence of ERG antibody (ERG) after immunoprecipitation (IP) using primers corresponding to indicated ERG putative binding sites (ChIP1 and/or ChIP2) of the each gene promoter. Molecular weight markers are shown on the left.

Additional file 13. Selected list of genes that are significantly changed after 3 days or 4of ES cell differentiation in the control versus ERG shRNA treated cells. The Fold change (FC)and Lower Bound of Fold change (LCB) of significantly changing genes is shown in BOLD.

References:

1. RC Perlingeiro: **Endoglin is required for hemangioblast and early hematopoietic development**. *Development* 2007, **134**:3041-8.
2. O Feraud, Y Cao, D Vittet: **Embryonic stem cell-derived embryoid bodies development in collagen gels recapitulates sprouting angiogenesis**. *Lab Invest* 2001, **81**:1669-81.
3. M Ramalho-Santos, S Yoon, Y Matsuzaki, RC Mulligan, DA Melton: **"Stemness": transcriptional profiling of embryonic and adult stem cells**. *Science* 2002, **298**:597-600.
4. EL Sonnhammer, G von Heijne, A Krogh: **A hidden Markov model for predicting transmembrane helices in protein sequences**. *Proc Int Conf Intell Syst Mol Biol* 1998, **6**:175-82.
5. C Li, WH Wong: **Model-based analysis of oligonucleotide arrays: expression index computation and outlier detection**. *Proc Natl Acad Sci U S A* 2001, **98**:31-6.
